# Are V1 Simple Cells Optimized for Visual Occlusions? A Comparative Study

**DOI:** 10.1371/journal.pcbi.1003062

**Published:** 2013-06-06

**Authors:** Jörg Bornschein, Marc Henniges, Jörg Lücke

**Affiliations:** 1 Frankfurt Institute for Advanced Studies, Goethe-Universität Frankfurt, Frankfurt, Germany; 2 Department of Physics, Goethe-Universität Frankfurt, Frankfurt, Germany; Indiana University, United States of America

## Abstract

Simple cells in primary visual cortex were famously found to respond to low-level image components such as edges. Sparse coding and independent component analysis (ICA) emerged as the standard computational models for simple cell coding because they linked their receptive fields to the statistics of visual stimuli. However, a salient feature of image statistics, occlusions of image components, is not considered by these models. Here we ask if occlusions have an effect on the predicted shapes of simple cell receptive fields. We use a comparative approach to answer this question and investigate two models for simple cells: a standard linear model and an occlusive model. For both models we simultaneously estimate optimal receptive fields, sparsity and stimulus noise. The two models are identical except for their component superposition assumption. We find the image encoding and receptive fields predicted by the models to differ significantly. While both models predict many Gabor-like fields, the occlusive model predicts a much sparser encoding and high percentages of ‘globular’ receptive fields. This relatively new center-surround type of simple cell response is observed since reverse correlation is used in experimental studies. While high percentages of ‘globular’ fields can be obtained using specific choices of sparsity and overcompleteness in linear sparse coding, no or only low proportions are reported in the vast majority of studies on linear models (including all ICA models). Likewise, for the here investigated linear model and optimal sparsity, only low proportions of ‘globular’ fields are observed. In comparison, the occlusive model robustly infers high proportions and can match the experimentally observed high proportions of ‘globular’ fields well. Our computational study, therefore, suggests that ‘globular’ fields may be evidence for an optimal encoding of visual occlusions in primary visual cortex.

## Author Summary

The statistics of our visual world is dominated by occlusions. Almost every image processed by our brain consists of mutually occluding objects, animals and plants. Our visual cortex is optimized through evolution and throughout our lifespan for such stimuli. Yet, the standard computational models of primary visual processing do not consider occlusions. In this study, we ask what effects visual occlusions may have on predicted response properties of simple cells which are the first cortical processing units for images. Our results suggest that recently observed differences between experiments and predictions of the standard simple cell models can be attributed to occlusions. The most significant consequence of occlusions is the prediction of many cells sensitive to center-surround stimuli. Experimentally, large quantities of such cells are observed since new techniques (reverse correlation) are used. Without occlusions, they are only obtained for specific settings and none of the seminal studies (sparse coding, ICA) predicted such fields. In contrast, the new type of response naturally emerges as soon as occlusions are considered. In comparison with recent *in vivo* experiments we find that occlusive models are consistent with the high percentages of center-surround simple cells observed in macaque monkeys, ferrets and mice.

## Introduction

Evolution and synaptic plasticity optimize the visual cortex for the processing of visual stimuli. The quantification of the degree of optimization has long been subject of theoretical and physiological studies. Among the most influential contributions are models such as independent component analysis [1]–[3] (ICA) and sparse coding [4] which became popular because they linked response properties of simple cells in primary visual cortex to the view of sensory systems as optimal information encoders [5]–. Since they were first introduced, many different versions of sparse coding and ICA have been investigated. While many technical studies focused on different ways to efficiently infer the model parameters [3], [9], many others investigated the assumptions used in the underlying stimulus model itself such as the sparsity prior or the assumed stimulus noise [10]–[13]. An assumption that has been investigated very little in the context of sparse coding models is the assumption of linear superposition of basis functions. For many types of data, linear superposition can be motivated by the actual combination of stimulus components (e.g., sound waveforms combine linearly). However, for image patches an assumption of linear superposition implies that component occlusions are not considered.

But does neglecting or including occlusions have an impact on receptive fields predicted by sparse coding? If so, what is the main difference if occlusions are considered and how do model predictions compare with experimental measurements? A critical inspection of standard sparse coding as a model for simple cell responses has recently been motivated by increasingly detailed experimental studies of simple cell responses. Using reverse correlation, a broad variety of receptive field shapes has been recorded, e.g., for macaque monkeys [14], ferrets [15] or mice [16]. In general, the distribution of receptive field shapes was found to be more diverse than the distributions predicted, e.g., by sparse coding or ICA [14]. The most significant qualitative difference from modeling predictions was the experimental finding of large numbers of simple cells with globular instead of Gabor-like receptive fields [14]–[16]. None of the seminal papers on simple cell coding [2], [17] had predicted such fields. Experimentally, globular fields were presumably not prominently reported earlier because of previously used estimation and/or cell selection methods. If oriented stimuli (often Gabors or light-bars) with different orientations and positions are used, cells with globular or center-surround fields are difficult to detect.

After the discrepancy of diverse receptive field shapes and standard encoding models was pointed out [14], further studies did show that large numbers of globular fields can be obtained in computational models [11], [18]–[20]. Notably, two of these models [11], [20] are sparse coding versions based on a linear superposition assumption. One uses a specific sparse prior and a specific hand-set sparsity [11]. The other [20] reports large numbers of globular fields for specific combinations of overcompleteness and sparsity. For the very large number of other studies on models with linear superposition (including all ICA models), no or only low proportions of globular fields were observed (compare, e.g., [9], [10], [21]).

**Figure 1:**
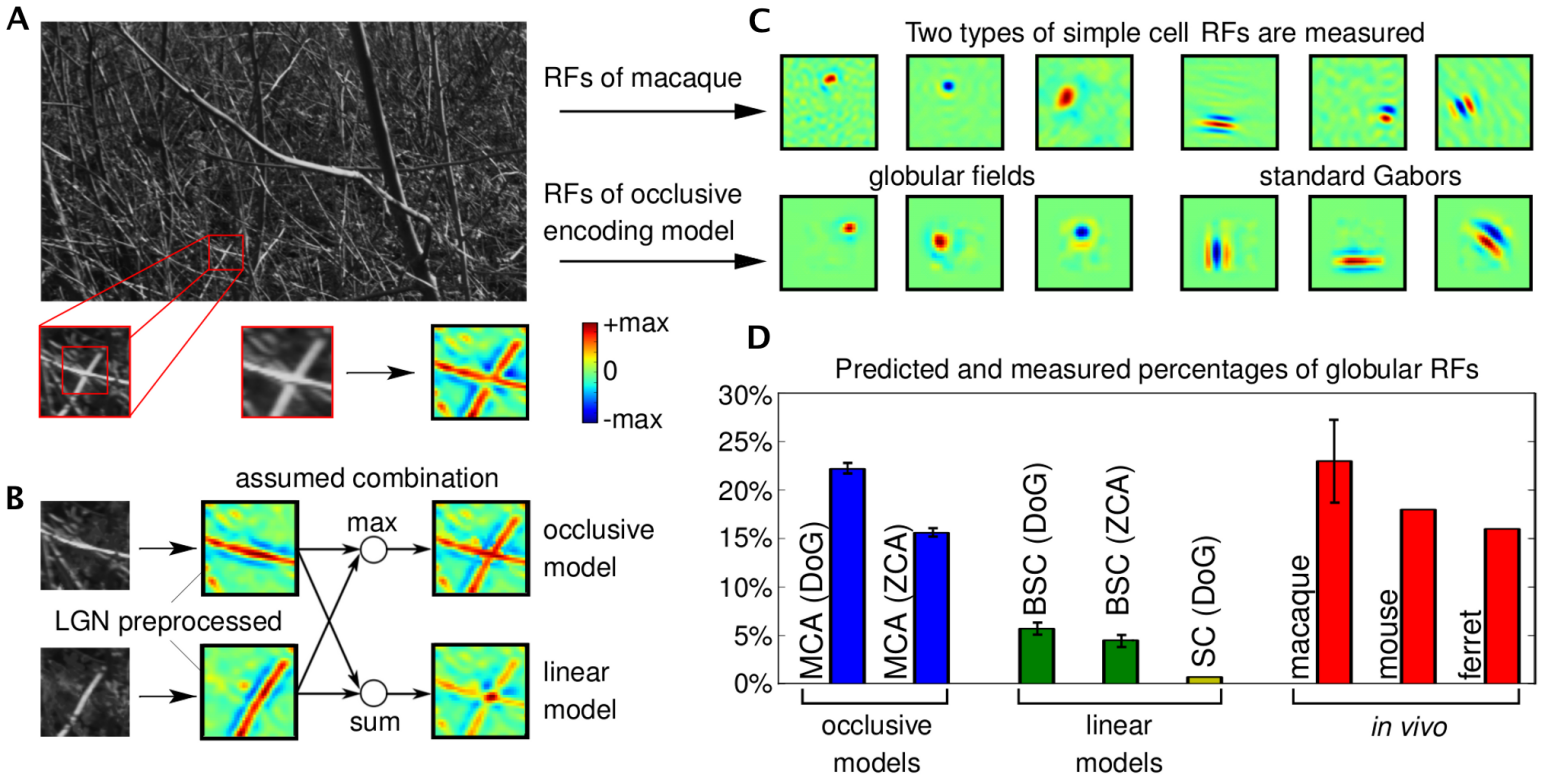
Illustration of the combination of image components, comparison with computational models of component combinations, and receptive field comparison. **A** Image patch (bottom left) showing an intersection of two branches extracted from a grey-level natural scene image (adapted from the van Hateren natural image database [58] with permission from J. H. van Hateren). Preprocessed version of the image patch (bottom right) obtained by using a center-surround filter to model the preprocessing by the lateral geniculate nucleus. **B** Left: Two image patches manually generated from the grey-level patch in **A**. Each patch is dominated by one of the two crossing branches of the original patch. Middle: The preprocessed versions of the two patches (central parts). Right: Combination of the two preprocessed patches using an occlusive combination (top) and a standard linear combination (bottom). **C** Examples of globular and Gabor-like receptive fields measured in V1 of macaque monkeys (courtesy of D. Ringach), and examples of the two receptive field types predicted by the occlusive encoding model. **D** Percentages of globular receptive fields predicted by different models for 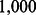 hidden units compared to percentages of globular fields of *in vivo* recordings.

**Figure 2:**
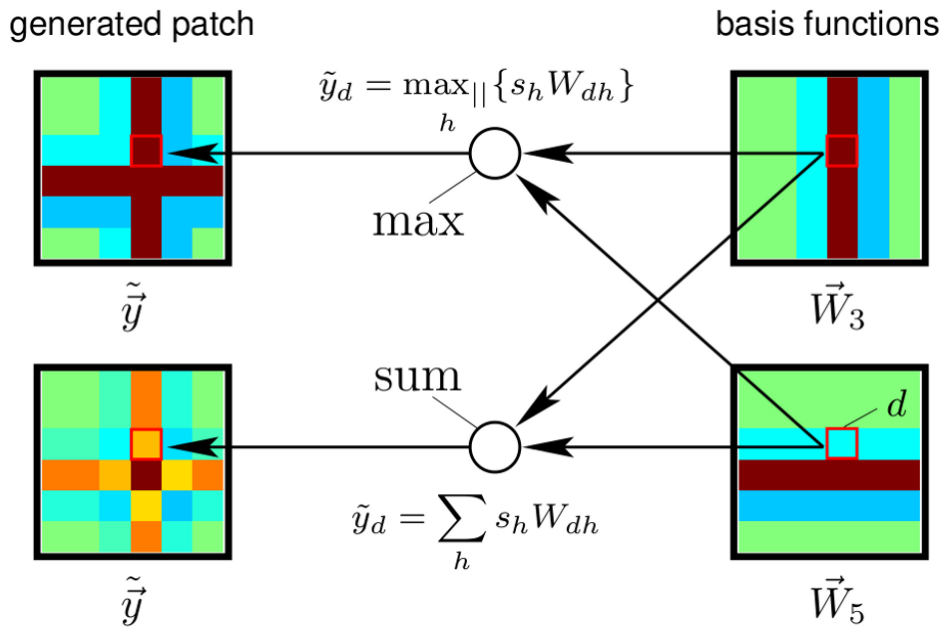
Example of the non-linear and the linear generative model. Suppose all hidden units are zero except of units 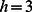 and 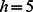. In this case the patch is generated using the basis functions 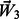 and 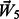. If the two basis functions have the form as displayed on the right-hand-side, the non-linear and the linear model generate the patches on the left-hand-side. Given a pixel 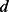, the non-linear model chooses the basis function 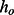 with the maximal absolute value (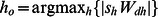) to set the value 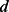 of the patch, 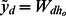. For the example pixel (red box), 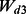 is chosen but for other pixels 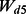 may be chosen. Note that the 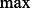-superposition models the exclusiveness of occlusion without considering object or edge depths. The linear model always sums the basis function values, 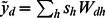. After the generation of the noiseless patches 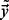 both models assume the addition of Gaussian noise for the generation of patches 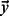 (see Eqns. 2 and 4 but not shown in the figure). The color scale is defined as in Fig. 1.

In this study we, for the first time, provide a systematic investigation of the impact of occlusion-like non-linearities on predicted simple cell responses. In order to quantify the differences to the neglection of occlusions, we study two sparse coding models: one assuming standard linear superposition [2], [17] and the other approximating occlusions with strongly non-linear superpositions of components [22], [23]. Fig. 1A,B illustrates the difference between the linear and the non-linear superposition used. By comparing the two combination rules with the actual combination of components in images, we can observe a better match of the non-linear superposition rule to the actual combination of components. If all components had the same intensity (i.e., the same color for the illustration in Fig. 1A,B), the 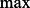-combination rule would represent the correct model for component occlusions [22] (also see Fig. 2). For components with different intensities, the non-linear combination is an approximation of the actual combination rule. However, the much weaker interferences resulting from the non-linear rule are a significantly closer match to occlusion non-linearities (see Fig. 1B).

Although the only difference between the two sparse coding models investigated is the rule for component combination, non-linear sparse coding versions have been investigated much less than linear versions because parameter optimization becomes more challenging. To model image patches for instance, large-scale applications of non-linear models with large numbers of observed and hidden variables have not yet been reported. By applying novel training methods [24] it is possible to overcome computational tractability limitations, e.g., for the strongly non-linear model illustrated in Fig. 1. Consequently, we can systematically study the effect of the combination rule on receptive fields predicted by sparse coding. The models' predictions will allow us to answer the question if and how occlusions can impact simple cell coding. Comparison of the model predictions to *in vivo* recordings then provides experimental evidence for the impact of occlusions on simple cell coding.

## Results

### Models for the encoding of image patches

We compare two generative sparse coding models for the encoding of image patches by simple cells. Both models have the same set of parameters and both assume, like standard sparse coding, independent visual components and Gaussian noise in the data. The distinguishing feature of the non-linear model is the use of a point-wise maximum to describe the combination of visual components. The maximum combination is illustrated and contrasted with the standard linear combination in Fig. 2. If we denote by 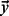 an observed image patch and by 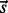 the hidden units encoding presence or absence of components, the full generative formulation of the non-linear model is given by:
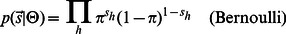
(1)

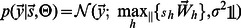
(2)This model is compared to one assuming the standard linear superposition:
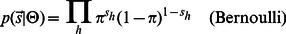
(3)

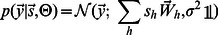
(4)The parameters of both models are the 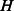 basis functions 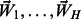 (which will later be related to receptive fields), the noise variance 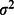, and the sparsity parameterized by 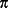. We define 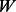 to be the matrix containing all basis functions (columns of 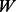) and for brevity denote 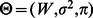 to be the set of all model parameters. The non-linear superposition in equation 2 is given by the function 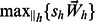 (compare Fig. 2). Instead of linearly summing basis function entries at pixel 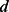 like in the linear model (Eqn. 4, 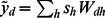), the mean value of the Gaussian, 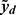, is set by the (active) basis function entry with maximal magnitude: 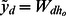 where 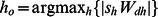. The function in (2) is the vector valued version defined by applying the maximum magnitude function for each entry. By using a point-wise maximum, the model is a variant of *maximal causes analysis* (MCA) [22], [23] and will be referred to accordingly. For the generation of image patches, both models assume a basis function to be either part of the patch or not (binary hidden variables). Such an assumption is consistent with objects or edges being either present or absent in a given patch. However, binary hidden units are different from conventional sparse coding in which continuous hidden variables are used. For later comparison, we therefore also study conventional sparse coding based on the generative model given by:
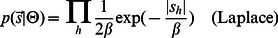
(5)

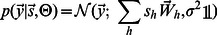
(6)where a Laplace prior is used to model continuous sparse values (instead of the Bernoulli prior used in the other two considered models). This model is the generative analogue of the objective function formulation of sparse coding with 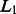 regularization. We will refer to the model of Eqn. 5 and Eqn. 6 as *standard sparse coding* (SC) and to the linear model with Bernoulli prior (Eqns. 3 and 4) as *binary sparse coding* (BSC) [25], [26].

For each model above we now seek the parameters that optimally model the statistics of image patches. As a result, each model predicts a set of basis functions which can be compared to each other and to *in vivo* recordings of simple cell receptive fields. To find optimal parameters, we apply maximum likelihood learning on the same set of preprocessed image patches (see Methods). For maximal causes analysis (MCA) and binary sparse coding (BSC) we applied a variational EM approach [24], while parameter optimization for standard sparse coding (SC) applied a maximum a-posteriori approach [4], [9]. All optimization approaches used allow for the inference of parameters for large numbers of input and hidden units. While large-scale applicability of linear sparse coding models has been demonstrated repeatedly in the past [9], [17], [27], comparatively efficient optimization of strongly non-linear models has only been demonstrated very recently [23], [24]. The optimization procedure applied to MCA and BSC furthermore allows the inference of all model parameters 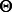 including stimulus noise and sparsity. The only remaining parameters are the size of image patches and the number of basis functions (with the degree of over-completeness given by the ratio of the two).

### Comparison of predicted receptive fields

**Figure 3:**
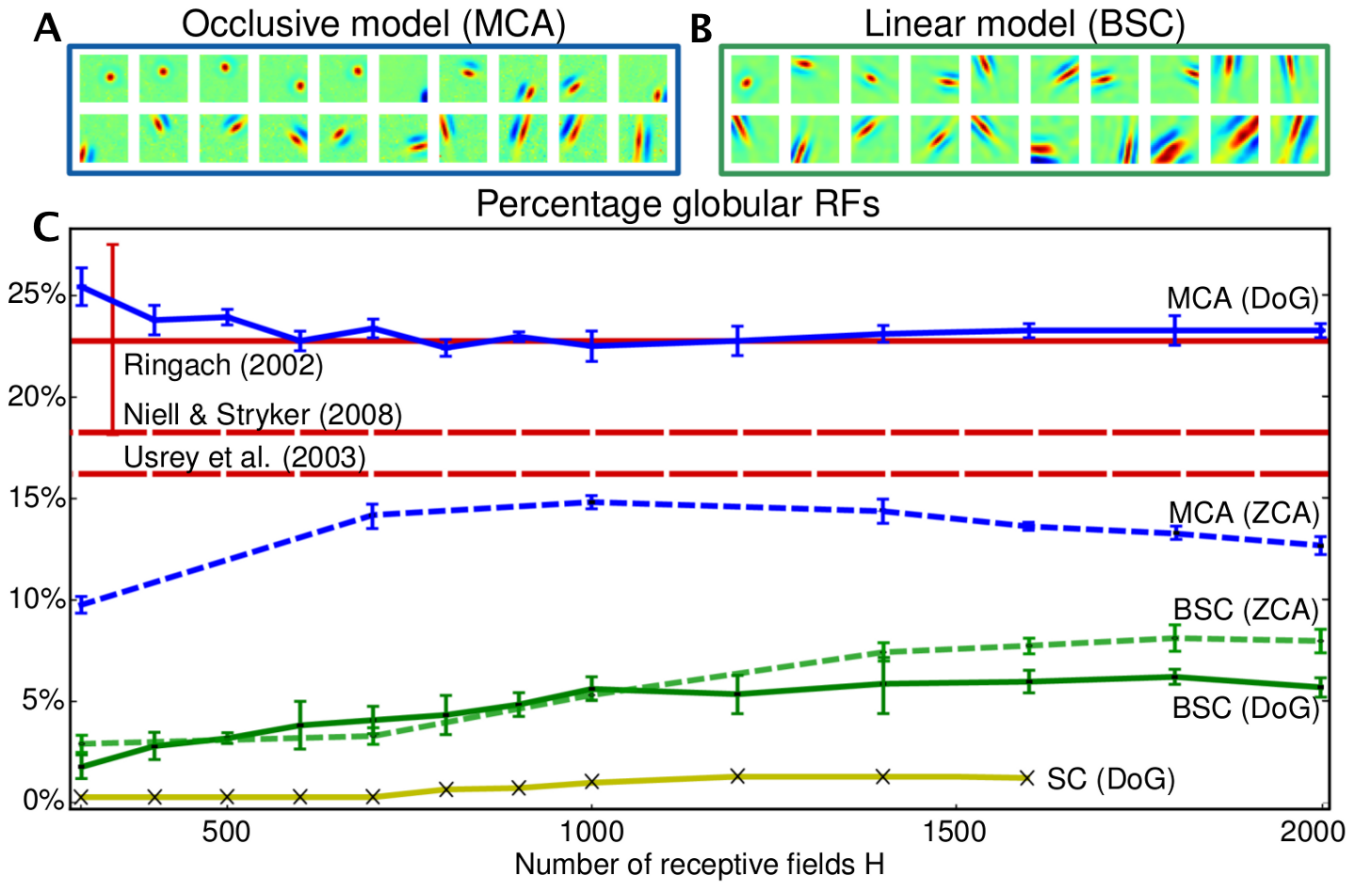
Percentages of globular receptive fields predicted by the computational models in comparison to *in vivo* measurements. **A** Receptive fields predicted if occlusion-like superposition is assumed (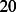 out of 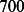 receptive fields are shown). **B** Receptive fields predicted if standard linear superposition is assumed (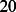 out of 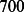 receptive fields are shown). **C** Percentages of globular fields predicted by the occlusive model (MCA) and by the linear model (BSC) versus number of hidden units. The experiments for MCA (blue line) and BSC (green line) on DoG preprocessed image patches were repeated five times and the error bars extend two empirical standard deviations. Standard sparse coding (yellow line) on DoG processed data shows the lowest fraction of globular fields. To control for the influence of preprocssing, additional experiments were performed on ZCA whitened data (dashed blue and dashed green lines). The bold red line (and its error bar) shows the fraction of globular fields computed based on *in vivo* measurements of macaque monkeys [14]. Dashed red lines show the fractions reported for ferrets [15] and mice [16].

For the generative models above, we optimized the model parameters for a set of natural image patches. First, natural image patches were preprocessed using an array of linear center-surround filters to model preprocessing by the lateral geniculate nucleus (LGN). Details are given in the Methods section. Given a fixed set of preprocessed stimuli, we optimized the parameters for the non-linear model (MCA), for binary sparse coding (BSC), and for standard sparse coding (see Methods and Supporting Information). All models were applied to the same set of preprocessed patches (no independent ON-/OFF-channels). After optimization, all models predicted a large number of Gabor-like receptive fields (compare Fig. 3 A,B). However, we found significant quantitative differences in the statistics of receptive field shapes. Most saliently, the different models showed different fractions of globular fields, i.e., fields that are not Gabor-like but are best described as center-surround (difference-of-Gaussians) fields [14]. In the primary visual cortices of different species, significant proportions of simple cells with such receptive fields have been reported [14]–[16] (see Fig. 1 C for examples of such cells in macaque monkeys). However, globular fields are either not observed or only done so in relatively small numbers when standard sparse coding or ICA are applied to image patches. We observed globular fields for both linear and non-linear models. However, the predicted proportions of such fields were very different. Fig. 1 D shows the proportions of globular cells for 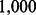 hidden units for the different models and Fig. 3 C shows the proportions for each model for different numbers hidden units (different degrees of overcompleteness). For standard sparse coding [9], the percentage of globular fields tends to increase corresponding to an increase in overcompleteness [27] but stays low in relative comparison (below 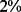).

Sparse coding with binary latents as in BSC results in a consistently higher percentage of globular fields ranging from 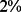 for 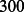 units to about 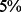 for 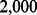. By far however, the highest percentages of globular fields were observed in applications of the non-linear model (MCA). Relatively independent of the number of latents, fractions between 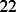 and 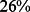 of globular receptive fields were obtained. For comparison, the fraction of globular fields in macaque monkeys [14] is estimated to be about 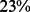 (see Methods and SI), in ferrets about 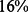 of the fields were reported to be globular [15], and in mice about 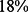 globular fields were measured [16]. For ferrets and mice the percentages were reported in the corresponding studies [15], [16], and for macaque monkeys we used original receptive field recordings (courtesy of D. Ringach) and applied the same classification procedure as for the predictions computed by the models (see Methods and Fig. S6 and S7). The percentages of globular fields estimated on the grounds of the three experimental studies [14]–[16] are given as horizontal red lines in Fig. 3 C.

**Figure 4:**
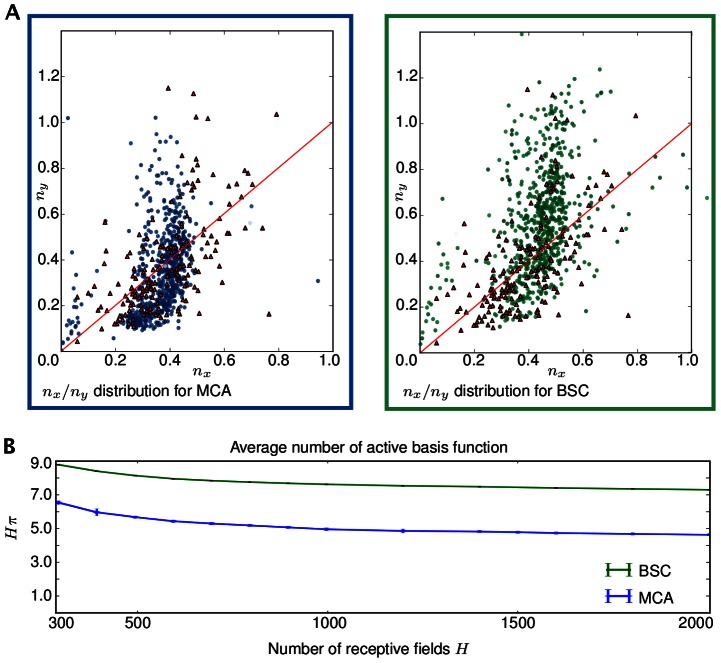
Comparison of Gabor shape statistics with *in vivo* recordings and predicted sparsity. **A** Analysis of learned Gabor-like receptive fields for experiments with 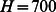 hidden units (and patch size 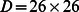): 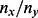 distribution of Gabor shaped receptive fields learned by occlusion-like (MCA) and linear sparse coding (BSC). The red triangles in both plots depict the distribution computed based on *in vivo* measurements of macaque monkeys [14]. **B** Average number of active units accross image patches as a function of the number of hidden units 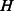 (note that error bars are very small; experiments on 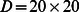 pixel sized DoG preporcessed patches).

Of all remaining non-globular fields predicted by the models, almost all have a Gabor-like shape (with few fields having unspecific shapes; see Methods and compare Figs. S3 and S7.). To analyze remaining differences between these Gabor-like fields, we followed an approach suggested by an earlier experimental study [14], i.e., we matched the fields with Gabor functions and plotted Gabor shape parameters (Gaussian envelope parameters and frequency) using dimensionless 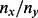-plots (see Methods and SI for details). 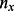 is proportional to the width of the Gaussian envelope in wave-vector direction; 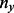 is proportional to its width orthogonal to the wave-vector. The widths are measured in multiples of the spatial wavelength. As we have separated out the globular fields first, we avoided having to match center-surround fields with Gabor functions, which removes a problem of earlier applications of the 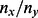 analysis. Fig. 4 A shows the obtained distributions for the non-linear and the linear model (for 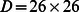, 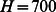), respectively. As can be observed, both distributions are relatively broadly shaped but differ. The distribution predicted by the non-linear model is shaped upwards starting from 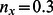 while the distribution predicted by the linear model is more elliptical. Furthermore, the receptive fields of the non-linear model tend to lie closer to the origin with a center-of-mass at about 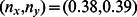 compared to a center-of-mass at 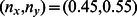 for the linear model. For comparison, we applied the same analysis of receptive field shapes to *in vivo* recordings of macaque simple cells [14] (data provided by D. Ringach, see Methods and Fig. S7). The resulting shape distributions are overlaid with the model predictions in Fig. 3 A. The center-of-mass of the experimental recordings lies at 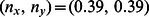 and is much closer to the center-of-mass of the non-linear model. In general, the distributions predicted by both models show a large diversity of Gabor shapes and a relatively large overlap with macaque recordings, however.

Other than investigating different models for image patch encoding, we explored different preprocessing methods prior to the application of the encoding models. We used a neurally plausible preprocessing by modeling LGN input to the cortex using center-surround (difference-of-Gaussians) filtered patches. Another (and related) method of preprocessing popular for functional modeling is zero-phase PCA whitening [28]. To control for the influence of the preprocessing method (i.e., the model for LGN input to the cortex), we applied the linear and non-linear models also to image patches preprocessed using zero-phase PCA whitening (ZCA). We found that preprocessing has a significant influence on the shapes of predicted receptive fields. A change in preprocessing both changes the percentages of globular fields (see Fig. 3 C, ZCA curves) and the shape distribution of Gabor fields (see Methods and Fig. S4). The main difference between the linear and non-linear receptive fields remains the consistently much higher percentage of globular fields for the non-linear model, however. Similarly, the degree to which center-ON and center-OFF cells are assumed to convey input independently from one-another [29] has an impact on the shapes of receptive fields. Controls with ON- and OFF-cells treated independently of each other again reproduce the same qualitative results, with the non-linear model showing a much higher percentage of globular fields than the linear model (see Fig. S5). Finally, also controls with sparsity levels fixed to the same values for both models always resulted in a much higher percentage of globular fields for the non-linear model. This much higher percentage was, without exception, observed in all of the experiments and controls of this study.

### Sparsity and inference

Unlike standard sparse coding [4] and most of its variants [9], [11], [30], the non-linear MCA model and the linear BSC model both do not only infer parameters for the basis functions but also parameters for sparsity and stimulus noise. Consequently, these parameters do not have to be hand-set or inferred by cross-validation in numerical experiments. More importantly, however, we can directly ask if the degrees of inferred sparsity differ between the non-linear and linear model. Sparsity is of high interest for understanding neural coding [31]–[33]. Theoretical predictions of sparsity levels have, so far, only been studied for linear models. Here we can study sparsity for the non-linear and linear model very directly. Because of binary hidden variables described by a Bernoulli prior, we use the number 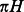 as sparsity measure. This number is simply the average number of active units across all image patches. Or in other words, the average number of basis functions a model needs to combine for the generation or reconstruction of an image patch. Note that the value 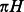 corresponds to an inverse sparsity (however, we will refer to this value as *sparsity measure* or simply *sparsity* if the meaning is clear from the context).

**Figure 5:**
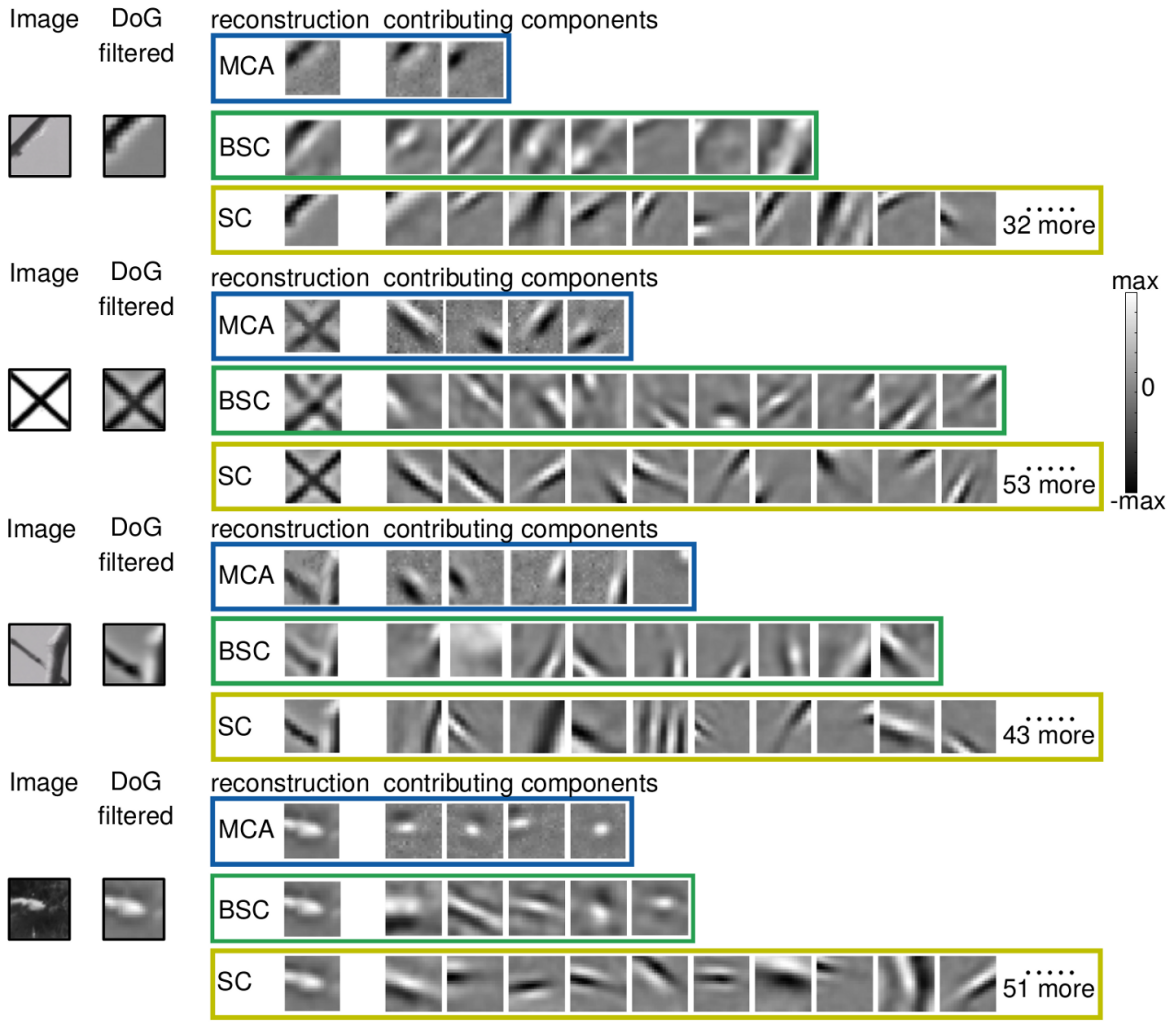
Decomposition of image patches into basic components for four example patches. For each example the figure shows: the original patch (left), its DoG preprocessed version (second to left), and the decomposition of the preprocessed patch by the three models. For better comparison with the original patches, basis functions are shown in grey-scale. The displayed functions correspond to the active units of the most likely hidden state given the patch. In the case of standard sparse coding, the basis functions are displayed in the order of their contributions. Standard sparse coding (SC) uses many basis functions for reconstruction but many of them contribute very little. BSC uses a much smaller subset of the basis functions for reconstruction. MCA typically uses the smallest subset. The basis functions of MCA usually correspond directly to edges or to two dimensional structures of the image while basis functions of BSC and (to a greater degree) of SC are more loosely associated with the true components of the respective patch. The bottom most example illustrates that the globular fields are usually associated with structures such as end-stopping or corners. For the displayed examples, the normalized root-mean-square reconstruction errors (nrmse) allow to quantify the reconstruction quality. For standard sparse coding the errors are (from top to bottom) given by 0.09, 0.08, 0.10 and 0.12, respectively. For the two models with Bernoulli prior they are larger with 0.51, 0.63, 0.53, and 0.42 for MCA, and 0.37, 0.47, 0.44 and 0.39 for BSC. We give reconstruction errors for completeness but note that they are for all models based on their most likely hidden states (MAP estimates). For MCA and BSC the MAP was chosen for illustrative purposes while for most tasks these models can make use of their more elaborate posterior approximations.

In analogy to Fig. 3 C, inferred degrees of sparsity are plotted in Fig. 4 B for different numbers of basis functions. For both models, MCA and BSC, the average number of active hidden units decreases (sparsity increases) with increasing number of basis functions (i.e., with increasing over-completeness). However, while both models converge to increasingly sparse solutions, the non-linear model was found to be consistently and very significantly sparser. On 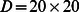 patches and 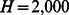 hidden variables the non-linear model estimates a patch to consists of on average four to five components (basis functions) compared to seven to eight as estimated by the linear model. Fig. 5 illustrates the different encodings of the two models for different example patches. For the simple example patch showing an oriented ‘branch’ (Fig. 5, top), both models combine basis functions of similar orientation. However, MCA uses fewer ‘line segments’ to re-construct the patch while BSC uses more basis functions. For patches with more complex structures (Fig. 5, examples in the middle), the differences become still more salient. Again, MCA uses fewer basis functions and usually reconstructs a patch from components which correspond to actual components in a patch. The final example (Fig. 5, bottom) illustrates inference with Gabor-like and globular components. The MCA model uses a globular field to reconstruct a two dimensional end-stopping structure. In the example, BSC reconstructs the patch by exclusively using Gabors. Some of them are very localized but clearly Gabor-like fields (the two right-hand-side fields). Often the BSC fields are not closely aligned with true image components. Sometimes we also observed BSC to use a globular field for an end-stopping structure but it does so much more rarely than MCA. We have never observed standard sparse coding to use a globular field for the examples investigated. In general, BSC and (much more so) standard sparse coding use more basis functions (reflecting the lower sparsity) and usually combine components which do not directly correspond to actual image components. In control experiments using different preprocessing approaches, we found that concrete sparsity levels do depend on the type of preprocessing. However, as was the case for the percentage of globular fields, in all experiments sparsity levels were consistently much higher for the non-linear model than for the linear one (see Methods and SI).

## Discussion

In this work we have investigated the impact of occlusion non-linearities in visual stimuli on simple cell coding. Specifically, we compared optimal coding of a linear sparse coding model to a sparse coding model taking strong occlusion-like non-linearities into account. The comparison of the two (otherwise identical) sparse coding models showed significant differences in the predicted receptive fields as well as in predicted levels of sparsity.

### Comparison of model predictions and *in vivo* recordings

The non-linear model consistently predicted a high percentage of globular receptive fields (Figs. 1 D and 3 C) which was relatively independent of the degree of overcompleteness (i.e., number of fields). The linear model and standard sparse coding showed much lower percentages. For comparison with *in vivo* recordings of simple cells, we used data from macaques [14], ferrets [15] and mice [16]. Notably, high percentages of globular fields were found in all these experimental studies. The percentage of globular fields in macaques was estimated here based on data provided by D. Ringach. By applying the same classification procedure as for the theoretical predictions, 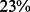 of the original receptive field recordings were classified as globular fields. For ferrets, 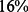 globular or *center-surround* receptive fields were reported [15]. For mice, 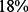 of recorded cells consisted of just one subfield [16], which is a close match to globular fields as defined in this work. It should be pointed out that none of the experimental studies had a focus on globular fields. These fields have been observed while general properties of V1 receptive fields were investigated.

For comparison, the experimentally measured percentages of globular fields (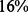, 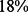, and 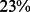) tend to be lower than the percentages predicted by the non-linear model (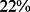 to 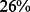) but they are much higher than the low percentages (below 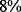) of the linear models. Fig. 3 C visualizes the predictions of the models for different degrees of overcompleteness with experimental results shown as horizontal lines. For the measurements and for the models, the percentages of globular fields can depend on different experimental or model settings. On the experimental side, receptive field measurements can depend, e.g., on the type of stimuli used for reverse correlation. On the modelling side, the percentage of globular fields can change, e.g., by changing sparsity levels or overcompleteness. For our comparative study we removed the arbitrariness in sparsity levels by applying an optimization procedure which automatically infers the level of sparsity. To study the influence of overcompleteness, we screened through different values for the number of hidden units. Considering all numerical experiments, the type of component superposition emerged as having by far the most significant influence on percentages of globular fields, with the non-linear model showing robustly very high percentages. Neither standard sparse coding with the usual parameter settings nor a range of other standard models predict such high percentages: For sparse coding, globular fields only emerge with specific priors and/or specifically chosen sparsity levels [11], [20], [30]. For independent component analysis, k-means, sparse auto-encoders or restricted Boltzmann Machines no globular fields were observed [21]. The high percentages of globular fields for the occlusive model studied here and the high percentages observed in *in vivo* recordings suggest a strong impact of visual occlusions on simple cell encoding.

Furthermore, the reported results suggest direct experiments to verify or falsify the models studied here: Suppose different simple cells with receptive fields at the same location in the visual field were identified, then the linear and non-linear models could be used to predict the responses if complex stimuli are presented at the same location. For a crossing of two edges the linear model would for instance predict responses less aligned with responses to the individual edges than the non-linear model (compare Fig. 5). This is because the linear model combines less specific components (and more of them) as they can be added and subtracted more freely than those of the non-linear model. The linear model would thus predict a higher difference between the response to overlapping line segments and the responses to the individual segments. Measuring the difference of a response to a crossing and to the individual lines would thus allow to verify or falsify the linear or non-linear model more directly. Also predictions of different sparsity levels could be verified or falsified but such experiments are more difficult because it is challenging to accurately measure sparsity levels *in vivo*. The consistently much sparser encoding predicted by a non-linear sparse coding model has, however, a significant potential impact on the ongoing debate on sparse encodings and recent experimental results [32], [33].

In contrast to differences in sparsity and in the percentage of globular receptive fields, we found the differences of Gabor-shape distributions (Fig. 4) less instructive for distinguishing image encoding based on linear or occlusion-like models. For both superposition assumptions we obtained a large diversity of Gabor shapes. Notably, both distributions are broader and have a larger overlap with macaque receptive fields than ICA and standard sparse coding [14]. As the non-linear and linear model studied here use binary hidden units, the higher overlap of both models with experimental results may, instead, be taken as evidence for a more discrete neural encoding of components than assumed, e.g., by a standard continuous Laplace prior [17], [27].

### Comparison to other computational models

Since the diversity of receptive field shapes was suggested as a means for comparison of models to experimental data [14], [34], different modeling approaches have been shown to result in broad distributions of Gabor shapes. Consistent with our observation that more discrete priors result in a large diversity of shapes, recent studies [11], [30] reported a large diversity based on more discrete values for the hidden units. Two studies [11], [20] notably obtained high percentages of globular fields by simultaneously assuming a linear combination of components. However, parameter optimization of both studies focused on the basis functions themselves, sparsity was hand-set and not inferred from data. One of the studies [11] specifically chose the sparsity level which resulted in the highest similarity between model and experimental distribution of receptive fields. The hand-set sparsities of these two linear models are, consequently, unlikely to be the optimal sparsity values for the data. It therefore remains an open question what percentages the models would predict for (approximately) optimal values of sparsity and data noise. For sparse coding with standard parameter settings (e.g., SC in Fig. 3 C), for novel linear sparse coding models (e.g., [30]) or for other models [21] no or only relatively few globular fields were observed. For the non-linear model investigated here, high percentages of globular fields robustly emerged in all experiments with sparsity levels (and data noise) always automatically estimated from the used set of image patches.

In addition to functional and probabilistic approaches to model simple cell coding, other computational investigations are based on models of neural circuits. While many studies directly relate to linear sparse coding [11], [30], [35], other contributions are not directly linked to an underlying functional model and, notably, often point out that non-linearly overlapping components can be learned well [19], [36]–[39]. The non-linear generative model studied in this paper can be seen as a functional correlate to neural circuit models that do well in learning non-linearly combining components. Consequently, a neural model for non-linear component extraction [19], [38] was among the first modelling approaches to report and discuss globular receptive fields [18], [19]. Such microcircuit models suggest that, on the one hand, a neural implementation of the non-linear model may have some advantages over the linear model because the 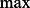-superposition is closely related to a (soft) k-winner-take-all competition or rank-coding among computational units [19]. On the other hand, standard linear models with appropriate sparse priors can be shown to result in mono-modal posteriors [17]. Such modes can efficiently be found using gradient-based neural dynamics which may represent a computational advantage of such models. In the case of ICA, activities of hidden units can directly be computed via filter responses.

In general there may, therefore, be relevant aspects other than the theoretical optimality of the generative model itself. To obtain as optimal as possible results, an encoding model has to fulfill two requirements: (A) it has to reflect the data generation process well and (B) it has to provide an efficient procedure to learn optimal parameters. A simpler model may in practice have the advantage of a more efficient learning procedure while learning based on a non-linear model may be harder. There may, for instance, be higher computational costs associated with a non-linear model or convergence to local optima may represent a problem. It has, therefore, been argued in the literature [40] that discussions about coding efficiency should contain learning efficiency as an integral part. In controls with our models using ground-truth stimuli, we indeed found a higher tendency of the non-linear model to converge to local optima compared to the linear model (see Methods, Numerical experiments). Learning still frequently converged to a global optimum, though, and could easily be improved using annealing. For natural image patches, we did not observe differences between runs with and without annealing (Methods). All experiments resulted in the same percentages of globular fields (within the limits of the error bars in Fig. 3C), for instance. Based on the used learning approach, finding optimal parameters therefore does not seem much more challenging for the non-linear model than for the linear one. Also the computational cost is about the same (compare Methods and [24]). Furthermore, both models face essentially the same challenges regarding neural implementability. Because of discrete hidden variables, a standard MAP estimation can not be applied and would be prohibitive for a direct inference of the optimal sparsity and stimulus noise. An implementation in neural microcircuits would consequently have to focus on how the posterior could be represented efficiently. This may be realized through population codes (e.g., [41], [42]) or through a sampling based representation (e.g., [32], [43]). The latter can be related to the approximation used here [44]. Accuracy and response times would then depend on the concrete realization of such a neural implementation. Functionally, sensory coding efficiency is very task dependent (see [40] for a discussion). Regarding metabolic coding efficiency, a sparser code is preferable over a less sparse code, which would favor the non-linear model. For image reconstruction, linear models may remain well suited (compare, e.g., reconstructions in Fig. 5), and a reduced sparsity can help for this task. However, best results for general tasks and for further processing in the visual pathway are presumably achieved for the best stimulus model, i.e., for a model which well approximates the actual stimulus generation process.

Note, that the maximum non-linearity and standard linear superposition as studied here are only two possible models for the combination of components. In the literature, other non-linearities such as noisy-OR combinations [45]–[47] or non-linear ICA [48] have been investigated before. Neither these non-linearities nor the maximum non-linearity have, so far, been shown to predict simple cell receptive fields, however. The reason is that non-linear models could, so far, not be scaled-up to the problem size required to study optimal codes on image patches. This is, again, due to the requirement of learning approaches that go significantly beyond MAP-based approximations.

Although sparse coding and its variants represent the standard model for simple cell coding, other computational models have been suggested. More recently, for instance, the suitability of mixture model approaches has been discussed [21], [49], [50]. While such models emphasize fitting model to data distributions, approaches such as ICA, sparse coding or MCA aim at learning a distributed encoding based on a combination of components. Still another functional approach to model visual stimuli is a line of research referred to as *dead leaves* approaches [50]–[53]. These statistical models of visual stimuli have long emphasized the importance of occlusions, and they were shown to reproduce many statistical properties of visual stimuli [52], [53]. So far, this prominent line of statistical image models was incompatible with sparse coding and simple cell models, though. The incorporation of occlusion non-linearities into sparse coding offers a way to reconcile these lines of research. Again it should be noted, however, that the non-linear model studied here accounts for occlusions by assuming strongly non-linear superpositions of low-level image components. A more explicit encoding of occlusion would result in a more accurate functional model but involves a larger set of parameters and further increases computational requirements [54]. Furthermore, explicit occlusion models are presumably more relevant for mid- and high-level vision (with objects and object parts as components) than they are for low-level image statistics.

### Why globular fields?

**Figure 6:**
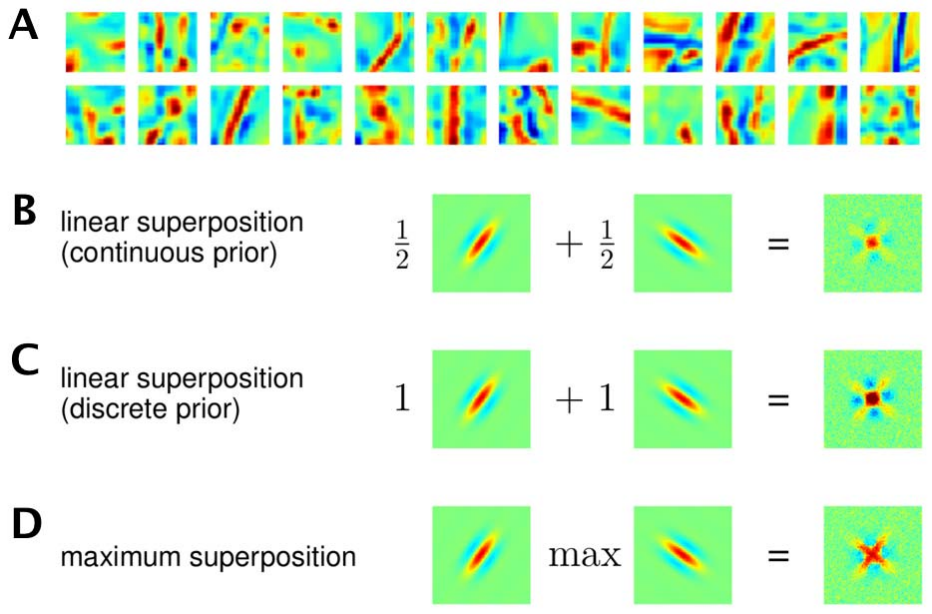
Illustration of different superposition models and globular fields. **A** Selection of typical preprocessed image patches. **B** Superposition of two Gabor fields as assumed by standard sparse coding with continuous priors (along with additive Gaussian noise after superposition). **C** Superposition of the same two Gabor fields if hidden units (prefactors) are binary. **D** Superposition of the Gabor fields if a point-wise maximum is used as superposition model.

While different recent models report that globular receptive fields do emerge in applications to image patches [11], [18], [19], [30], they offer no explanation *why* this is the case. In this context, our comparative study allows for an explanation that is closely linked to discrete hidden units and the superposition model. First consider the selection of typical DoG preprocessed image patches as displayed in Fig. 6 A. As can be observed, the patches contain Gabor-like components as well as globular components. Also note that the maximal intensities of Gabor and globular components are similar. Now suppose that a sparse coding model has already represented Gabor-like fields such as those shown in Fig. 6 B (left-hand-side). If these two Gabor fields are linearly superimposed and then rescaled by a factor 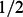 (Fig. 6 B), an (approximately) globular field is generated. If the two Gabors are linearly superimposed but can not be rescaled (Fig. 6 C), then the intensity of the globular field becomes higher than the intensity of typical globular structures in the data. For the non-linear superposition (Fig. 6 D) no globular structures can be generated by superimposing Gabors. Fig. 6 illustrates that globular structures in image patches can be explained by linearly superimposing Gabors. For linear sparse coding approaches with continuous values for hidden variables, globular structures do, consequently, not have to be represented explicitly. This may explain why almost all versions of sparse coding or ICA do not predict globular fields or only very low percentages thereof [2], [3], [9], [10]. If hidden units are prevented from taking on continuous values [11], [26], a stronger incentive is generated to explicitly represent globular fields. This can explain the observation of larger numbers of globular fields for models with more discrete priors [11], [26], [30]. A strongly non-linear superposition of Gabors can not generate globular fields. Consequently, such components have to be represented explicitly. This may explain the high percentages of globular fields in the non-linear model and, presumably, the high percentages of globular fields in the experimental measurements. Also note that the generation of globular structures in the linear models requires more fields than in the non-linear model, which is consistent with the sparser encoding in the non-linear case.

Both Gabor-like and globular fields are useful for image encoding. While Gabors are closely associated with edges, we observed globular fields to be more closely associated with two dimensional structures (see Fig. 5) such as corners or ends of branches (also compare [20] for a discussion). Furthermore, both component types may be useful for texture encoding. Both types are certainly observed in preprocessed stimuli (Fig. 6 A) and they are both measured *in vivo*. On the functional side, many tasks seem to work well with approaches *not* resulting in globular fields, as a large body of literature, e.g., on image processing with linear models shows. Also inference examples, e.g. those of Fig. 5, show that linear models (with low percentages of globular fields) can perform well, e.g., in terms of image reconstruction (mainly because they use a large number of components which they can add and subtract). For data with non-linearly combining components, non-linear models are naturally performing better if inference of the true components is the task [22], [24], [38], [55]. The functional capabilities of non-linear models and globular fields will, therefore, be very task dependent. The observation that globular fields are observed in *in vivo* recordings may, however, be interpreted as evidence for them being functionally very useful for the typical tasks animals and humans have to accomplish.

### Conclusion

Our study answers whether occlusions can have an impact on theoretical predictions of simple cell models. Based on a direct comparison of superposition assumptions we have observed very significant differences between the receptive fields and sparsity levels predicted by the linear and the occlusive model. Both models represent approximations of the exact model for local visual component combinations. However, we have observed that a non-linear superposition results in both a closer match to the true combination rule of visual components and a closer match of predicted receptive fields to *in vivo* measurements. This higher consistency between predicted receptive fields and *in vivo* recordings suggests that stimulus encoding in V1 is optimized by taking visual occlusions into account. Most significantly, high quantities of a new type of simple cells with center-surround fields, reliably and robustly emerge if visual occlusions are considered.

## Methods

### Optimization of model parameters

In this study we compared the predictions of two sparse coding models, MCA and BSC, when trained on natural image patches. Given the generative models (Eqns. 1 and 2 for MCA; Eqns. 3 and 4 for BSC) and a set of preprocessed image patches 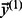 to 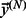 we sought for each model the parameter values 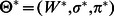 that maximize the data likelihood. In its logarithmic form the likelihood function is given by:
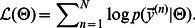
(7)For all models considered here (MCA, BSC and conventional SC), the optimization of the likelihood function represents a computationally intractable problem for higher dimensional hidden spaces. We therefore require approaches that approximately but efficiently optimize the likelihood. For MCA and BSC we apply variational expectation maximization [56] (variational EM). That is, instead of maximizing the likelihood directly, we maximize the so-called free-energy:
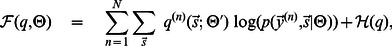
(8)where the sum 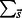 runs over all binary vectors 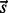 and where 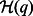 is an entropy term. The free-energy function 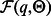 is a lower bound of the log-likelihood. By applying variational EM, the function is maximized alternately with respect to 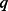 in the E-step (while 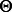 is kept fixed) and with respect to 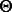 in the M-step (while 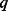 is kept fixed). For the M-step, expectation values of functions 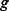 with respect to distributions 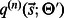 have to be computed. The optimal choice for these distributions in the E-step are the posterior probabilities given the stimulus, 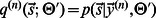. Sparse coding models are computationally intractable because these exact posterior distributions and their expectation values are intractable.

*E-step*. To efficiently optimize the models' parameters, we apply a variational EM approach by choosing distributions 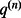 which are truncated approximations to the exact posteriors [24]:
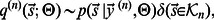
(9)where 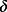 is an indicator function (i.e., 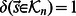 if 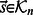 and zero otherwise) and where 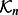 is a data point dependent subset of the hidden space. By choosing the variational distributions 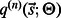 as in Eqn. 9, we obtain the following approximations for expectation values with respect to the exact posteriors:
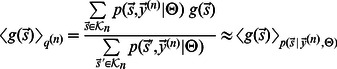
(10)The sums for the approximate expectation values now run over 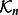 instead of the entire hidden space. If 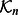 is chosen to be small but to contain the states with most posterior probability mass, the computation of the expectations in Eqn. 10 becomes tractable while a high accuracy of the approximations is maintained [24]. The set 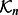 is, therefore, chosen to consider the subset of the 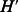 most relevant hidden units for a patch 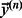. Furthermore, at most 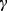 of these 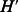 units are assumed to be active simultaneously 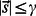. More formally we define:
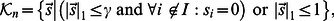
(11)where the index set 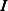 contains those 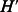 hidden units that are the most likely to have generated data point 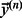 (while the last term in Eqn. 11 assures that all states 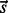 with just one non-zero entry are also considered). To determine the 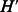 hidden variables for 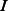, we use those units 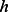 with the 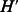 largest values of a *selection function* 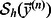 given by:
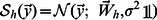
(12)Through the selection of states with high posterior mass, the function resulted in a high accuracy for parameter recovery on data with ground-truth (see numerical experiments further below). Parameters of the approximation are the maximal number of components considered, 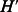, and the maximal number of simultaneously active components 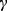. They can be chosen such that a high approximation accuracy is achieved with simultaneously high efficiency (see numerical experiments).

*M-step*. If the variational distributions 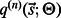 of the free-energy are chosen as in Eqn. 9, then M-step equations for parameter updates follow from the optimization of a truncated free-energy [24] which is given by:
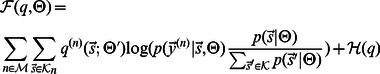
(13)where 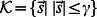 is the set of all states with less than 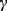 active hidden units. The set 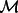 is a subset of those data points with less or equal 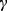 components. Data points with more than 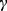 components are not well approximated and are therefore not considered for learning. 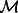 is defined to contain the 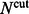 data points with smallest values for 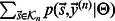, where 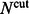 is the expected number of well approximated data points [24] given by 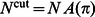 with 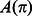 defined as in Eqn. 20 below.

#### MCA update equations

The M-step equation for the generative fields 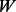 for MCA is derived along the same lines as for the original MCA model [22], [24]. However, the scalable algorithm in [24] did not infer data noise 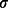 nor data sparsity 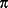. Furthermore, note that the MCA model used in this work applies a point-wise maximum *magnitude* function. Instead of being aimed at positive data as the original MCA algorithm, the maximum magnitude version developed for this work is directly applicable to data with positive and negative values, and it treats (like sparse coding) these values equally. The model is, therefore, directly applicable to the same data as standard sparse coding or BSC. Additional channel separation [23], [57] to convert preprocessed stimuli to positive values is consequently not required, which reduces the difference between MCA and BSC to the component combination rule alone.

To derive update equations for 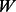 we first replace the 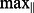 operation by a smooth approximation 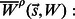


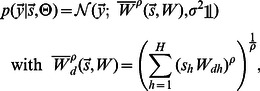
(14)where 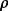 is a large and odd positive integer. Note that in the limit of 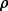 approchaing infinity, 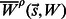 becomes the 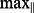 operation we replaced it for:
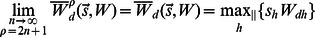
(15)To maximize the truncated free-energy 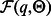 (Eqn. 13) with respect to 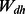, we use equation 14 and obtain:
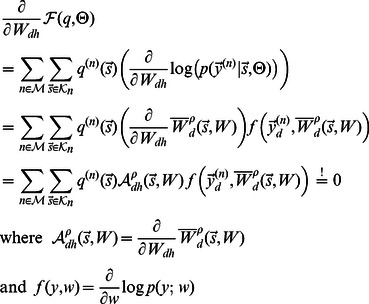
(16)with 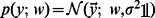. Now, for any well-behaved function 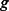 and for large values 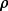 we can write
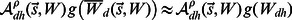
(17)because 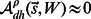 whenever 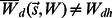. Hence it follows from Eqn. 16 that:
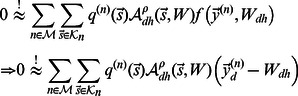
(18)Rearranging terms of (18) results in the update equation for 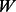 (see Eqn. 23 below).

The derivation of the M-step update for 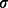 is straight-forward. The derivation of the M-step for 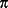 involves a term that corrects for discounting the data points with more than 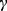 components. This term is a consequence of the additional prior term in the truncated free-energy (Eqn. 13). For the derivation we used
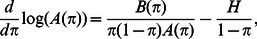
(19)with
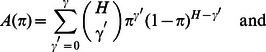
(20)

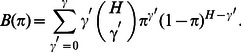
(21)By taking the derivative of the truncated free-energy (Eqn. 13) with respect to 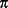 we then obtain:
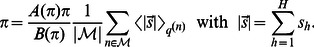
(22)Applying this equation in the fix-point sense (compare Eqn. 25) results in a convergence to values 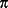 that represent solutions of Eqn. 22.

To summarize, the M-step equations for the MCA model are given by:
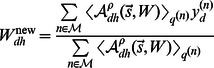
(23)

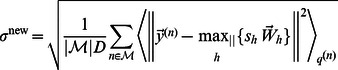
(24)

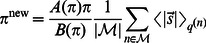
(25)where 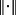 in Eqn. 24 denotes the 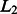-norm. Eqns. 23 to 25 with expectation values as given in Eqn. 10 represent the learning algorithm of the MCA generative model.

One important property of the max-function of the MCA model is that only the largest value of its arguments determines the function's value. In the case of a finite dataset for optimization, this has the effect that those elements of the matrix 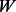 with small absolute values, have an influence on only very few of the supplied data points 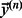. In these cases the updated values for 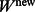 (Eqn. 23) are, therefore, based on very low evidence from the data. At the same time, with the maximum-function, even small changes to 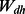 can change which basis function is responsible for a given data point element 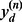. As a result, many close-to-zeros elements 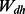 frequently change their value in an EM iteration. While their values stay close to zero, the exact values irregularly vary with each EM iteration due to the finite size of the dataset. To address this effect, we introduced a learning rate 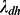, which slows down the learning for those basis functions that only have low evidence:
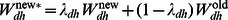
where we set 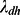 to be a monotonous function between 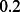 and 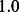 based on the amount of evidence that was available for each of the matrix elements 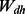:
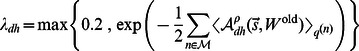
The reasoning behind this choice is that for each data point 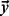 the expectation value 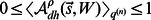 quantifies the responsibility of elements 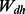 for explaining the data point. With this choice, the learning rate is 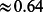 when a matrix element is responsible to explain only two data points, while it rapidly approaches 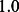 when it is responsible for explaining more than 10 data points. This modification insures numerical stability due to finite sample sizes without biasing the optimization result.

The computational complexity of the MCA learning algorithm is dominated by the number of states that have to be evaluated for each E-step. The scaling of this number can be estimated to be (compare [24]):
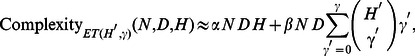
(26)where 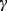 and 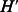 are the approximation constants introduced earlier. The first term is associated with the preprocessing step, the second with the combinatorics of the selected units. 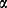 and 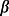 are scaling constants. They depend on the computational costs of the concrete functions for preselection and state evaluation.

#### BSC update equations

For the BSC model, the derivation of the M-step for 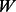 is analogous to the derviation of 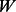 for standard sparse coding (and other linear models). The M-step for the data noise 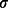 is straight-forward, and the derivation for the M-step for the sparsity parameter 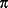 is analogous to the corresponding derivation of the MCA model. The resulting M-step equations are given by:
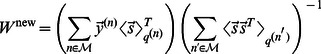
(27)

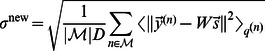
(28)

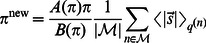
(29)where the set 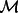 is defined as above. Because of the standard linear superposition used by the BSC model, the update equation of 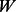 has the same form as for standard sparse coding (or principal component analysis). The only difference is the summation over the subset 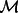 instead of the whole set of data points. The update equation for the data noise 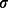 is the same as for MCA except of the combination rule, while the M-step equation for the sparsity 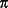 is identical to the one for MCA (but note that the distributions 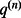 are different due to the different generative models). Likewise, the computation of the expectation values is analogous to MCA and uses the same definition of 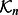, the same selection function, and the same values for approximation parameters 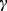 and 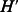. Accordingly, the computational complexity of the BSC learning algorithm is essentially the same, with the difference of a smaller scaling factor 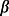 in Eqn. 26.

#### Parameter initialisation

For all numerical experiments with MCA and BSC the model parameters needed to be initialized. We used the same initialization procedure for both models and set the basis functions 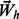 to the data mean plus Gaussian noise (unit variance), the sparsity parameter to correspond to one active component on average (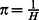) and the data noise 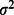 was set to the variance of the data:
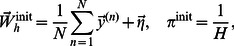
(30)

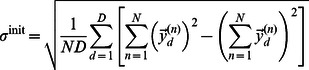
(31)All the source code and the datasets to rerun our experiments are publically available at: http://fias.uni-frankfurt.de/~bornschein/NonLinSC

#### Parameter optimization for conventional SC

For standard sparse coding we applied a MAP based approximation to optimize the parameters 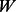. All experiments were run using a publically available implementation which is based on an earlier publication [9]. We used the standard 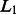 sparsity function and set the batch size to 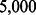. The number of bases 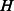 was set according to the experiment while parameters in the code (e.g., 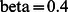) were left unchanged. For alle experiments, the algorithm detected to have reached an optimum after about 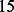 iterations. For small 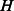 we performed 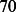 iterations but did not encounter any more changes after an optimum was detected. For 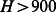 we thus only ran 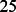 iterations. Computational demand became impractically large for experiments exceeding 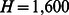.

### Numerical experiments - artificial data

To verify that the learning algorithms for MCA and BSC correctly recover data components at least approximately, we first applied them to artificial stimuli where ground-truth is available. For each model, a dataset of 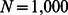 stimuli 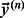 was generated. The generation followed the MCA and BSC model, respectively, using the same set of generating parameters for the basis functions, stimulus noise and sparsity. The used stimuli consisted of patches with 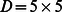 pixels generated from ten basis functions in the form of horizontal and vertical bars (five bars for each orientation). The parameter values of each bar were defined to be either 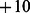 or 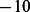 (with small amounts of additive Gaussian noise). The generating sparsity was set to 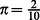 (two bars on average), and the stimulus noise was set to 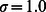. Examples of the generated patches are shown in Fig. S1 A for the MCA model, and in Fig. S2 A for the BSC model. The stimuli represent forms of a standard ground-truth stimulus set [36]. For MCA experiments the 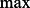 softening parameter 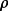 in equation 14 was set to 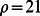 (a large odd integer). The MCA and BSC algorithms were run on the respective data using 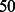 EM iterations each. For both algorithms the first third of the iterations (up to EM step 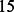) were performed on the full dataset with 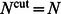. For iterations 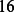 upto 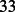 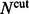 was linearly decreased to 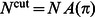. After 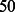 EM iterations, both models recovered the generating parameters of the data with high accuracy. The recovered generative fields after 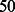 iterations and the time courses of data noise and sparsity are shown in Fig. S1 B–D for the MCA model, and in Fig. S2 B–D for the BSC model. Parameter optimization for both models is non-convex but, after convergence, we observed the parameters to represent the ground-truth basis functions for both models in most of the trials. MCA we observed to converge more frequently to local optima. By applying annealing, MCA and BSC both more efficiently avoided local optima. The bars stimuli have very pronounced local optima because the stimulus values are not continuously distributed. For stimuli with more continuous distributions of observed values such as images, we observed no significant differences between runs with and without annealing. In particular, no significant differences in the numbers of globular fields were observed. Both algorithms were, therefore, run without annealing for all the experiments on image patches.

### Numerical experiments - natural image patches

To optimize the model parameters on natural image stimuli, we extracted a set of 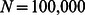 patches of size 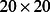 pixels for one set of experiments, and 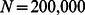 patches of size 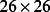 for another set of experiments. Patches were extracted at random positions from the van Hateren natural image database [58]. In mammals, visual information is transferred to the visual cortex via center-ON and center-OFF cells in the lateral geniculus nucleus (LGN). The sensitivity of these neurons can be modeled by a difference-of-Gaussians (DoG) filter. We therefore preprocessed all patches by convoluting them with a difference-of-Gaussians kernel. Following experimental results [59], the ratio between the standard deviation of the positive and the negative Gaussian was chosen to be 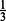 and the amplitudes were chosen to obtain a mean-free center-surround filter [19], [23]. After DoG filtering, values were scaled to fill the interval [−10,10] which provides a form of divisive contrast normalization [60]. Control experiments with divisive variance normalization [28], [60] (which serves the same purpose) produced closely matching results. To control for the influence of the DoG convolution filtering, we ran further experiments using zero-phase PCA whitening (ZCA) which represents a standard preprocessing procedure often used with functional models [28]. Furthermore, we controlled for the influence of separating positive and negative channels.

For each experiment, the same set of stimuli was used to train the three models under consideration. Those experiments, where we screened through different degrees of overcompleteness (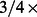 overcomplete with 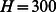 to 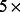 overcomplete with 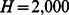) were performed on 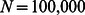 stimuli of size 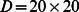 pixels (Fig. 3 C and Fig. 4 B). Each experiment was repeated five times to obtain empirical error bars on the recovered sparseness and the predicted percentage of globular fields (we show twice the standard deviations in Figs. 3 C and 4 B). All other experiments, including those investigating the 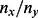 shape statistics (Fig. 4 A) were performed on 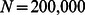 stimuli of size 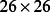. In total, results of 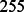 experiments were gathered to create Figs. 3 C and 4 B; additionally, about 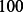 experiments were performed for various 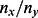-plots and for additional controls on differently preprocessed sets of image patches (see below). For each experiment on image patches we performed 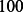 EM iterations. Analogously to the verification experiments on artificial data, the first 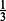 of the EM steps (1 up to 33) were run on the full dataset. For iterations 34 to 66, 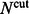 was again linearly decreased to 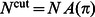 and kept at 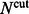 for the last 34 EM steps. The smoothing parameter for the non-linearity of the MCA algorithm was set to 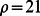 as for the artificial data. The approximation parameters for the non-linear and the linear model were both set to 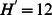 and 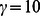. Each experiment to find optimal parameters was typically run on 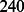 CPU cores using a parallelized implementation.

#### Controls for different LGN models

To control for changes of receptive field shapes depending on different types of preprocessing, we applied MCA and BSC to zero-phase PCA (ZCA) whitened patches [28] and to DoG preprocessed patches with an independent treatment of center-ON and center-OFF fields.

ZCA: Zero-phase PCA (ZCA) preprocessing is common in more technical applications of sparse coding or ICA. We replaced the DoG convolution by ZCA and normalized the patches as for DoG preprocessing. When MCA and BSC are applied to ZCA whitened data, the globular field percentages change with a lower percentage of globular fields for MCA as one consequence. Also for ZCA whitened data, globular field percentages for MCA remain consistently and significantly higher than for BSC (with at least 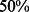 more globular fields for MCA; compare Fig. 3 C, dashed blue and green lines). Also the shape distribution of Gabor-like receptive fields changes: we observed for both models more fields elongated along the wave-front, i.e., higher 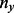 values (compare Fig. S4 B). This increase in elongation is somewhat more pronounced for the BSC model than for MCA.

Independent ON-/OFF-channels: In mammals, visual information is transferred to the cortex via two types of neurons in the lateral geniculus nucleus (LGN): center-ON and center-OFF cells. ON- and OFF-cells project to the primary visual cortex (mainly layer 4). Pairs of center-ON and center-OFF cells can be combined to provide a net center-surround input to cortical cells. Such ‘push-pull’ inputs are suggested by strongly overlapping receptive fields of LGN cells connecting to the same cortical column (see, e.g., a recent study [29] for discussions and references). We modeled such inputs by using DoG preprocessed patches for numerical experiments. However, center-ON and center-OFF inputs to the cortex may also be assumed to be entirely independent a-priori. The model for this latter situation would correspond to a separation of negative and positive inputs after DoG preprocessing. To control for the effect of independent ON and OFF inputs, we considered experiments on patches that are DoG preprocessed and normalized as above except of a subsequent separation into inputs for positive and negative parts. More formally, we used the same DoG filter and preprocessing to generate patches 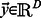 as previously but then converted them into patches 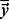 of size 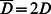 by assigning: 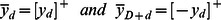 (for 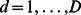) where 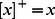 for 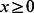 and 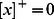 otherwise (see Fig. S5A for an illustration). Note that 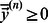 holds after separation. As a consequence the 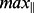 for the MCA model (Eqn. 2) reduces to the conventional 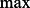 function. The applications of MCA and BSC to DoG preprossed image patches assuming independent ON- and OFF-cells essentially reproduced the results for the previous DoG preprocessed patches. Exemplarily, using 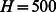 fields, we find that (1) BSC used, on average, more active units to encode a given image patch than MCA (Fig. S5C); (2) MCA inferred a much higher fraction of globular receptive fields than BSC (Fig. S5C); (3) MCA and BSC resulted in different distributions of Gabor field shapes (Fig. S5D). The differences in the 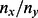-distributions is again not very pronounced, however.

In general, the type of preprocessing has an impact on the shapes of predicted receptive fields - affecting both percentages of globular fields and Gabor shape statistics. However, the difference in the percentages of globular fields with a consistently much higher percentage for the non-linear model is a very stable observation for all used preprocessing models. Also the sparsity of the non-linear model has always been observed to be much higher. Differences between the non-linear and linear model were much less pronounced if the shape distributions of Gabor-like fields were considered. While we found differences between the models for different preprocessing types, they were small compared to differences in sparsity and globular field percentages. At the same time, all distributions using 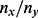-plots show a large diversity of fields with relatively large overlap with *in vivo* recordings. The analysis of 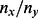-distributions has by now frequently been applied to analyse the quality of simple cell models [11], [18], [19], [30], [61] but for the purposes of this study we found 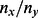-distributions much less instructive than percentages of globular fields and sparsity levels.

### Analysis of receptive fields

After parameter optimization we computed an estimate of the predicted receptive fields by convolving the learned basis functions 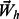 with the same DoG filter as used for preprocessing. Subsequently, we matched both the predicted receptive fields and the *in vivo* data with Gabor-wavelets and difference-of-Gaussians to gather the statistics of shapes.

The convolution with the DoG filter is an estimate of the receptive field assuming a linear mapping: If 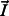 denotes a patch (with pixel values as vector entries) and if 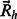 parameterizes the mapping, the linear response is given by 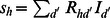. The original response of a unit to a patch consists of two steps: a linear preprocessing and a non-linear response to the preprocessed patch, where the non-linear response is described by the corresponding sparse coding model. We therefore rewrite the mapping 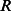 as a two-step mapping. If 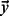 denotes a preprocessed patch (as in the main text), it is given by:
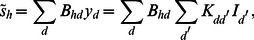
(32)where 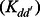 is the DoG kernel for the convolution and where 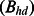 parameterizes a linear mapping from preprocessed patches to hidden units. The mapping 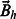 can be estimated by reverse correlation [14] using the models' approximate posteriors as responses. If we denote such an estimate by 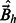, the total linear response is given by:
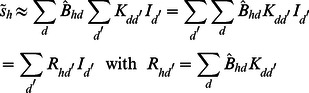
(33)This means the receptive field estimate is given by 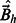 convoluted with the same kernel as used for preprocessing. Fig. S6 (top row) shows examples of estimates obtained in this way. Alternatively, note that the basis functions 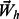 are already similar to stimuli that best drive the hidden units. A direct estimate of the parameters 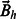 is therefore given by the basis function parameters themselves (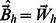), and the corresponding receptive field estimate is given by convoluted basis functions: 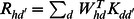. In numerical experiments, both estimates resulted in very similar receptive fields, and some representative examples are shown in Fig. S6. Because of this high similarity we used the convoluted basis functions as receptive field estimates, which reduced the otherwise extensive computational costs of reverse correlation for the very large number of receptive fields that were analysed in this study.

To analyse the shape statistics of the estimated receptive fields resulting from our numerical experiments and from experimental recordings [14], receptive fields were matched against Gabor-wavelets 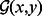 and difference-of-Gaussians 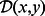. Note that for notational purposes we replace the index 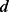 denoting the input units by two-dimensional coordinates 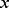 and 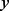 denoting the actual planar position in the two-dimensional field. The *in vivo* data analysed for comparison was obtained in experiments on macaque monkeys in an earlier study [14]. These receptive fields were recorded from neurons in the primary visual cortex using reverse correlation, and were matched with Gabor and DoG functions in the same way as the receptive fields predicted by the models. Representative examples are shown in Fig. S7 A. For each receptive field 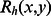, we sought the eight parameters which minimized the mean squared error between the field and the Gabor-wavelet 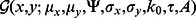. Where 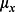 and 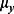 are the center coordinates of the Gabor-wavelet, 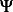 is its spatial rotation, 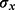 and 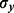 parameterize the shape of the Gaussian envelope, 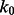 is a measure of the frequency of the planar wave component, 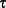 is its phase shift and 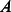 is the overall amplitude of the Gabor-wavelet:
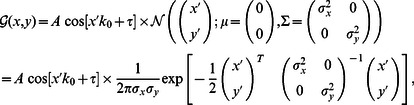
(34)where 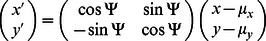 are the translated and rotated coordinates of the function.

Similarly, again for each receptive field 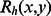, we sought the eight parameters of the difference-of-Gaussians kernel 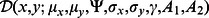 which minimized the squared distance to each field. 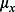 and 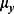 are the center coordinates of the DoG kernel, 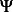 its spatial rotation. 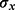 and 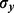 parameterize the shape of the inner Gaussian, 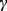 parameterizes the size difference between the Gaussians and 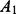 and 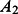 specify the amplitudes of the Gaussians:
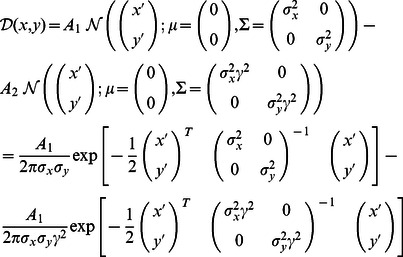
We classified a receptive field as being globular if the reconstruction error of the best matching DoG function was smaller then the reconstruction error of the best matching Gabor wavelet and if the aspect ratio of the DoG was smaller than 2.0 (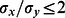, where 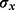 is the parameter for the more elongated axis). A small difference between the errors of a match with DoG and a match with a Gabor function means that the receptive field is neither clearly center-surround nor clearly Gabor-like. In such cases we call the field *ambiguous*. Using a standard least-square optimization method [62], we got robust result for fitting and classification for almost all receptive fields. We applied matching and classification to the results of each of our numerical experiments as well as to the experimental data [14] provided by D. Ringach. The experimental data consisted of 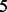 fields of 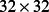 pixels, 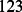 fields of 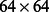 pixels, and 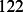 fields of 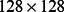 pixels. Our procedure classified 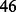 fields as clearly globular and 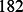 as clearly Gabor-like (see Fig. S7 A for some examples). As the experimental data is less smooth than the theoretical receptive field predictions, a relatively large number of 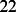 (out of 250) fields were ambiguous in this case (see Fig. S7 B for some examples). By considering half of these fields as globular, we obtained 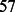 globular fields (a percentage of 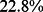); considering all of them as globular corresponds to 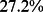 globular fields; and considering all ambiguous fields as Gabor-like results in a percentage of 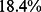 globular fields. In Fig. 2 C we used 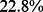 as mean with the higher and the lower percentages defining the limits of the corresponding error bar.

To analyse the shape distribution of receptive fields, the shape relevant parameters can be visualized as an 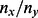-plot. That is, for each receptive field (predicted or measured) the dimensionless values given by 
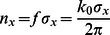
 and 
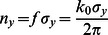
 were computed, where 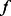 is the spatial frequency of the fitted Gabor function, and where 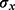, 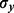 are the standard deviations of its Gaussian envelope in wavevector direction and orthogonal to it [11], [14], [19], [30]. For our analysis, we first removed the globular fields from the sets of experimentally measured fields as well as from the sets of predicted receptive fields before visualizing the corresponding 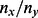 distributions. This procedure removed the otherwise ill-posed problem of having to match center-surround fields with Gabor wavelets.

## Supporting Information

Figure S1
**Experiments with MCA on artificial data.** **A** Random selection of 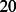 artificially generated data points with basis functions in the form of bars. Each data point 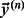 is composed of 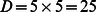 pixels. **B** Learned basis functions 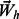. **C, D** Evolution of the inferred sparsity 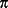 and the noise parameter 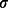 over a course of 50 EM steps (dashed lines indicate ground-truth).(TIFF)

Figure S2
**Experiments with BSC on artificial data.** **A** Random selection of 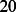 artificially generated data points with basis functions in the form of bars. Each data point 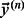 is composed of 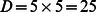 pixels. **B** Learned basis functions 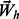. **C, D** Evolution of the inferred sparsity 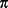 and the noise parameter 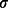 over a course of 50 EM steps (dashed lines indicate ground-truth).(TIFF)

Figure S3
**Example results when applying MCA and BSC to DoG preprocessed images.** **A** Predicted basis functions for MCA (left) and BSC (right) with 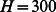 hidden units each. **B** Predicted basis functions for MCA (top) and BSC (bottom) with 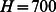 hidden units each.(TIFF)

Figure S4
**Results when applying MCA and BSC to zero-phase whitened data (ZCA).** **A** Full set of learned basis functions when applied with 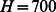 hidden units. **B** Distribution of shapes for the Gabor-like fields in **A**.(TIFF)

Figure S5
**Results when MCA and BSC are applied to DoG preprocessed data with independent ON- and OFF-center channels.** **A** Visualization of the doublication of input dimensions for independent ON and OFF channels. **B, C, D** Results for MCA and BSC after running on 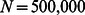 patches (size 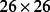 pixels) with independent ON and OFF channels. The number of hidden variables was set to 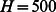.(TIFF)

Figure S6
**Comparison of receptive field estimates.** Representative examples of receptive fields estimated from basis functions 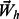 are shown. Estimates based on reverse correlation (top row) are shown together with their corresponding estimates based on direct convolution of the basis function (bottom row).(TIFF)

Figure S7 **Fitting of learned and** ***in vivo*** **receptive fields with Gabor functions and DoGs.** **A** Selection of 16 of the 250 receptive fields measured in macaque monkeys [15] using reverse correlation together with their resulting matches. **A** The upper row shows original recordings that were classified as globular, and the second row shows the corresponding DoG matches. The third row shows original recordings that were classified as Gabor-like, and the forth row shows their corresponding matches. **B** Examples of original receptive fields that were ambiguous, i.e., neither clearly difference-of-Gaussian nor Gabor-like. Note the Gaussian fields can be well matched by DoG and Gabor functions and are therefore inherently ambiguous. **C** A selection of 16 receptive field estimates resulting from numerical experiments. The fields and their matches are shown as in **A**.
(TIFF)
