# Supplementary figures and images for: Are V1 Simple Cells Optimized for Visual Occlusions? A Comparative Study
Source: PLoS Comput Biol. 2013 Jun 6;9(6):e1003062. doi: 10.1371/journal.pcbi.1003062 (PMC3675001; doi:10.1371/journal.pcbi.1003062)

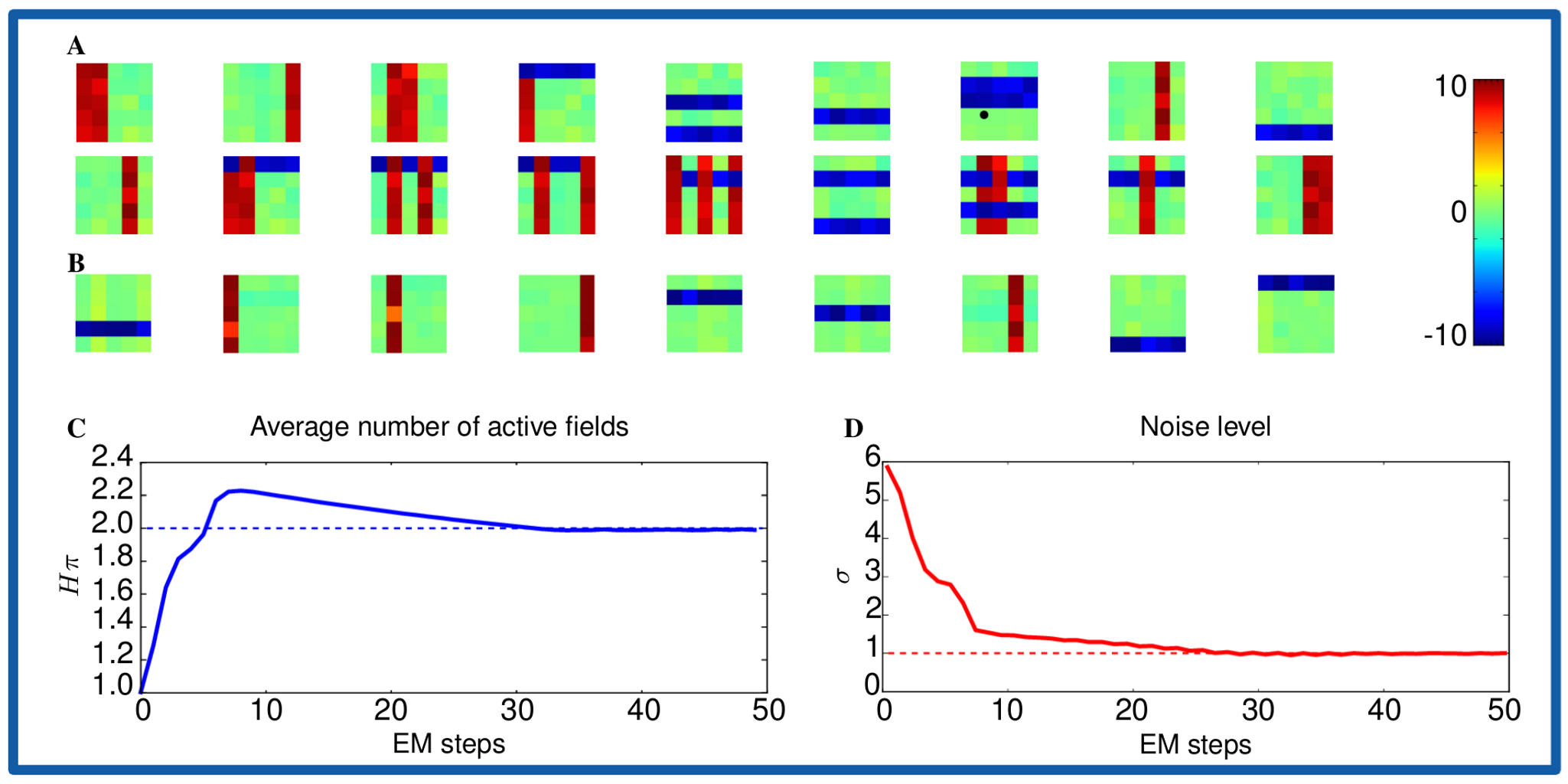

Supplement: Figure S1 — Experiments with MCA on artificial data. A Random selection of artificially generated data points with basis functions in the form of bars. Each data point is composed of pixels. B Learned basis functions . C, D Evolution of the inferred sparsity and the noise parameter over a course of 50 EM steps (dashed lines indicate ground-truth). (TIFF) [file pcbi.1003062.s001.tif]

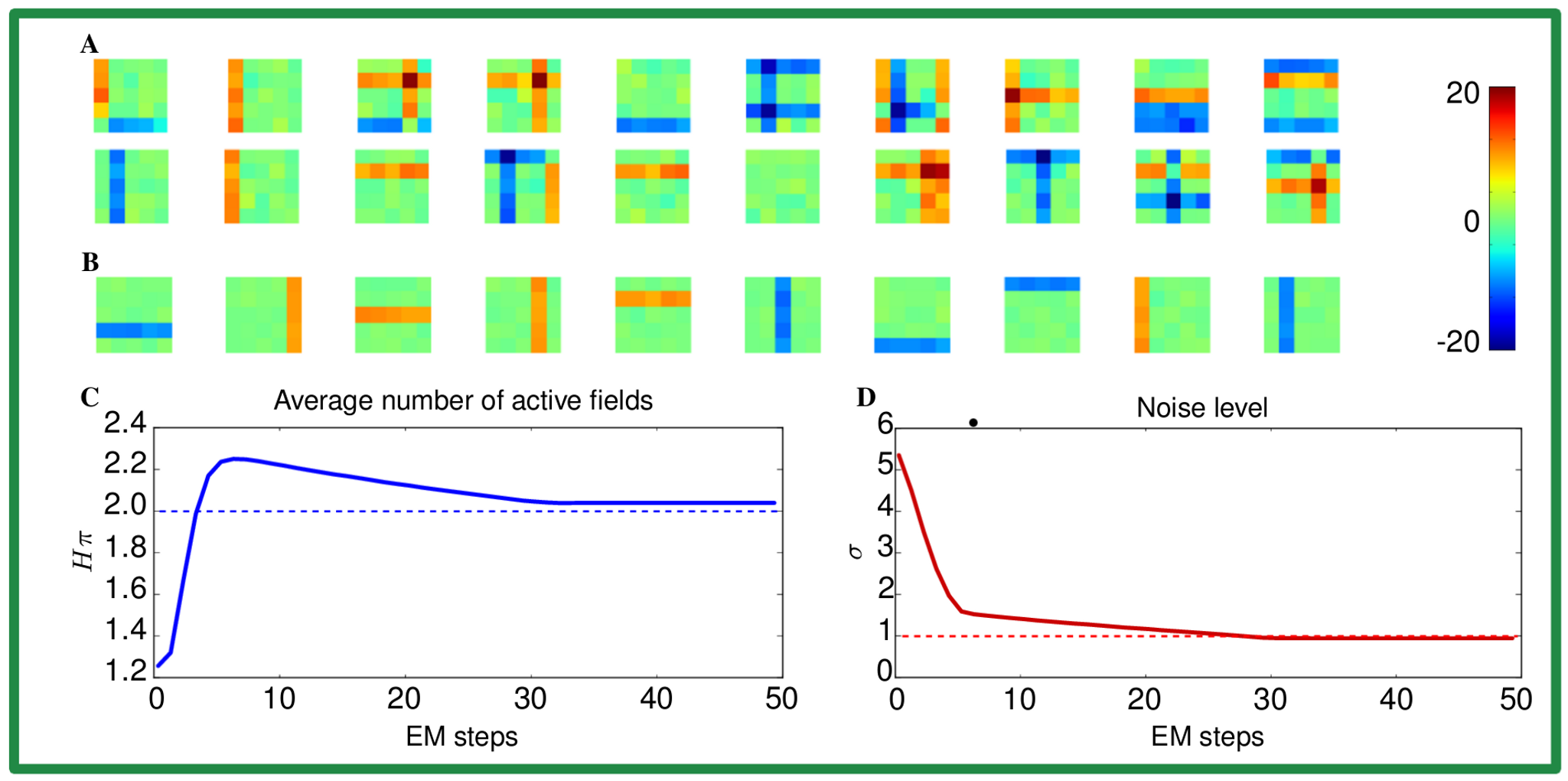

Supplement: Figure S2 — Experiments with BSC on artificial data. A Random selection of artificially generated data points with basis functions in the form of bars. Each data point is composed of pixels. B Learned basis functions . C, D Evolution of the inferred sparsity and the noise parameter over a course of 50 EM steps (dashed lines indicate ground-truth). (TIFF) [file pcbi.1003062.s002.tiff]

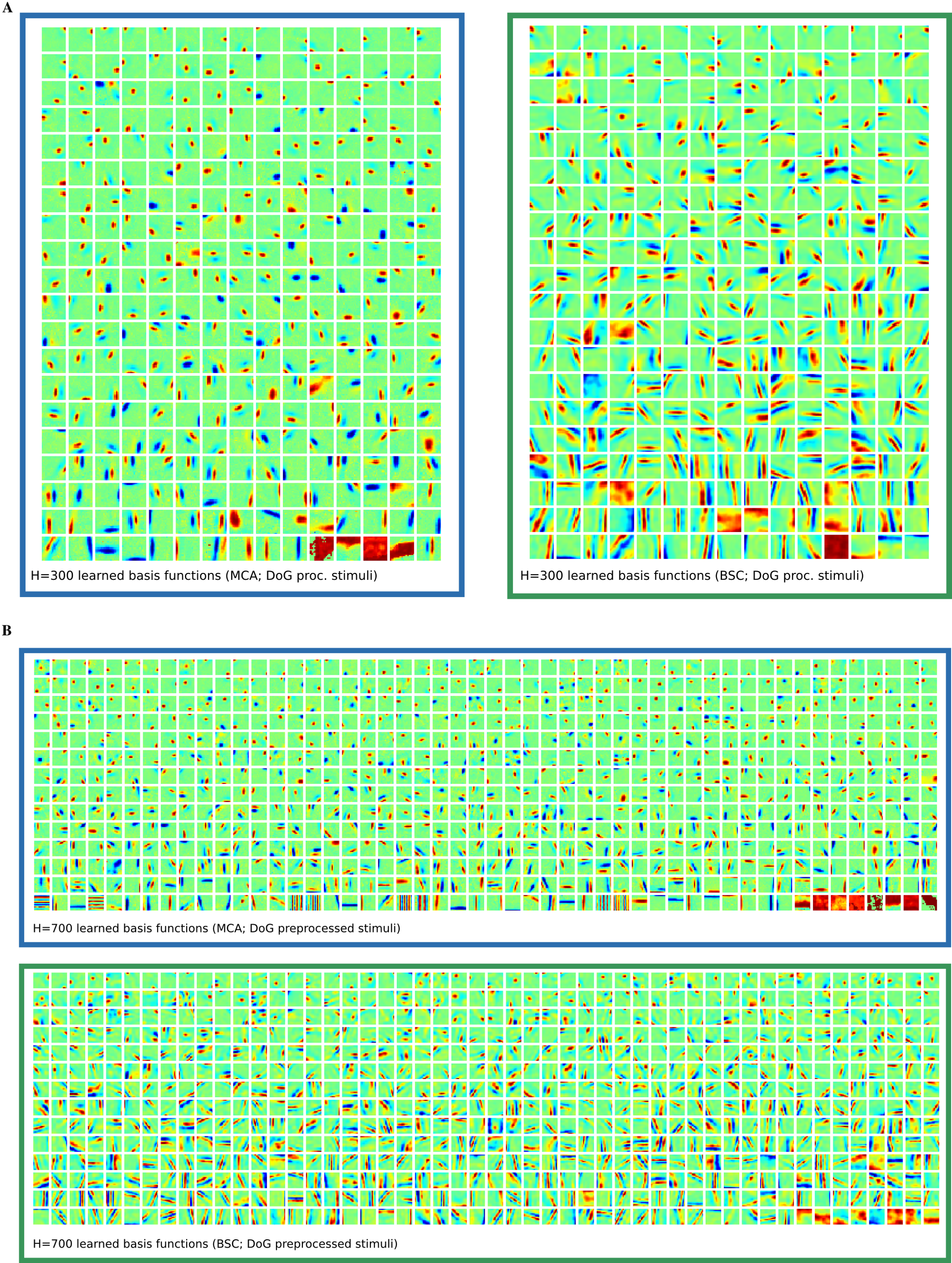

Supplement: Figure S3 — Example results when applying MCA and BSC to DoG preprocessed images. A Predicted basis functions for MCA (left) and BSC (right) with hidden units each. B Predicted basis functions for MCA (top) and BSC (bottom) with hidden units each. (TIFF) [file pcbi.1003062.s003.tiff]

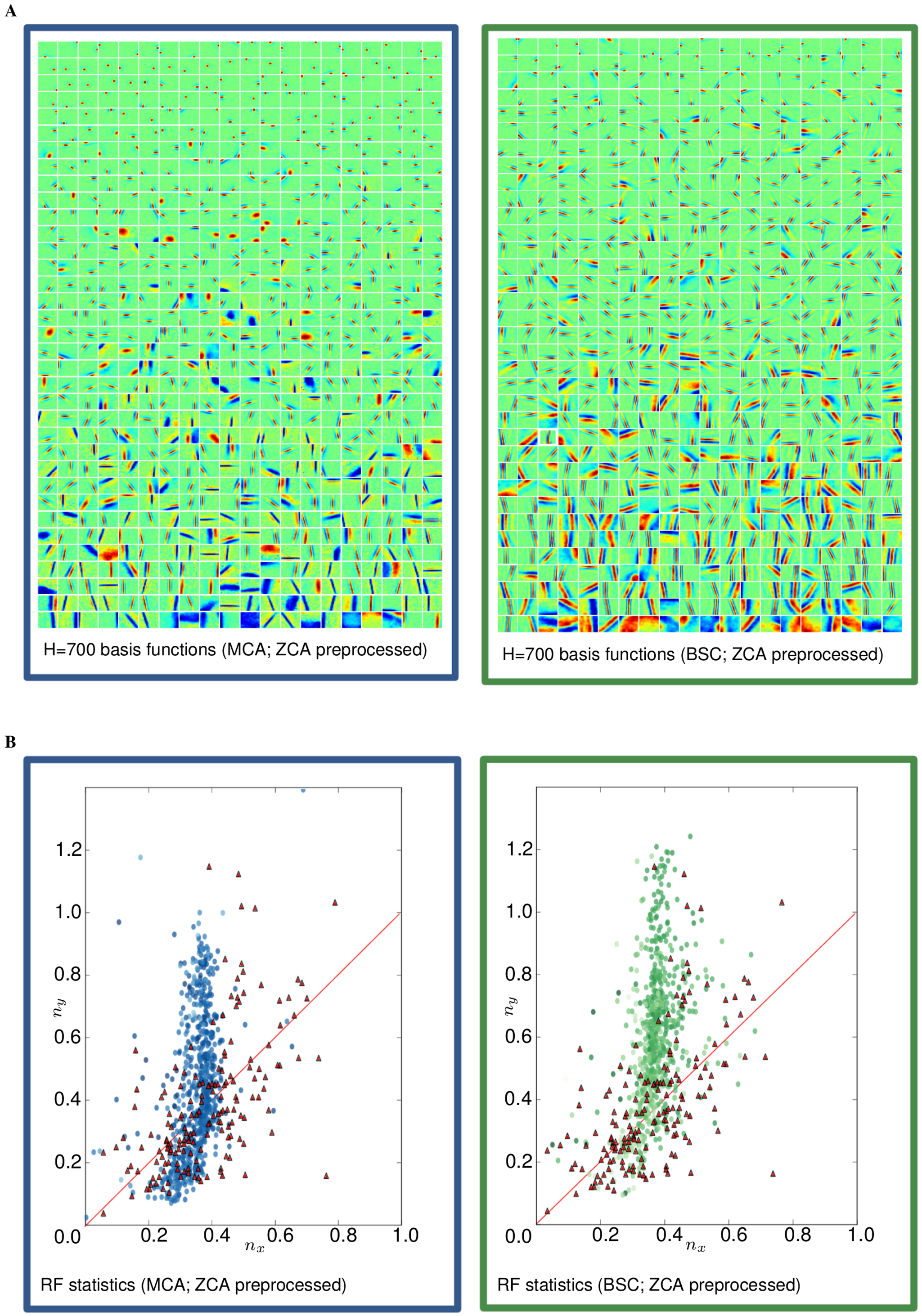

Supplement: Figure S4 — Results when applying MCA and BSC to zero-phase whitened data (ZCA). A Full set of learned basis functions when applied with hidden units. B Distribution of shapes for the Gabor-like fields in A. (TIFF) [file pcbi.1003062.s004.tiff]

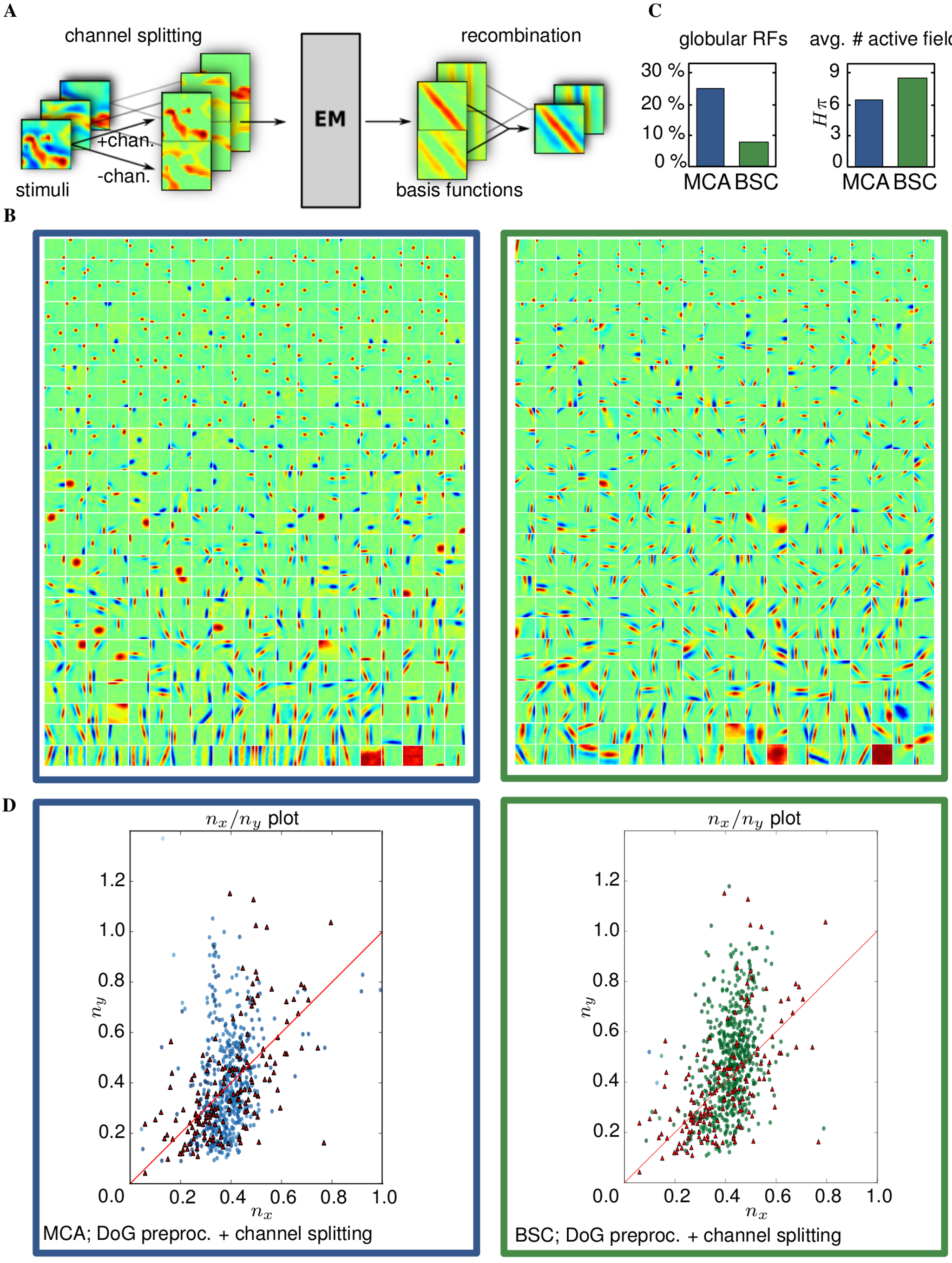

Supplement: Figure S5 — Results when MCA and BSC are applied to DoG preprocessed data with independent ON- and OFF-center channels. A Visualization of the doublication of input dimensions for independent ON and OFF channels. B, C, D Results for MCA and BSC after running on patches (size pixels) with independent ON and OFF channels. The number of hidden variables was set to . (TIFF) [file pcbi.1003062.s005.tiff]

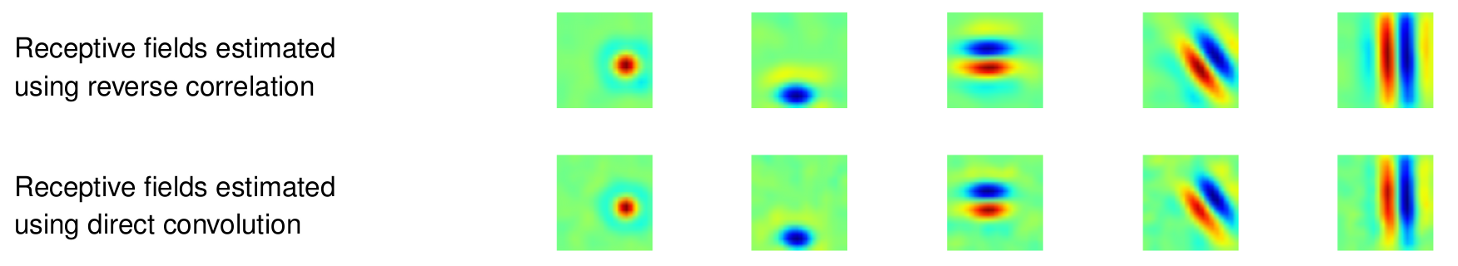

Supplement: Figure S6 — Comparison of receptive field estimates. Representative examples of receptive fields estimated from basis functions are shown. Estimates based on reverse correlation (top row) are shown together with their corresponding estimates based on direct convolution of the basis function (bottom row). (TIFF) [file pcbi.1003062.s006.tiff]

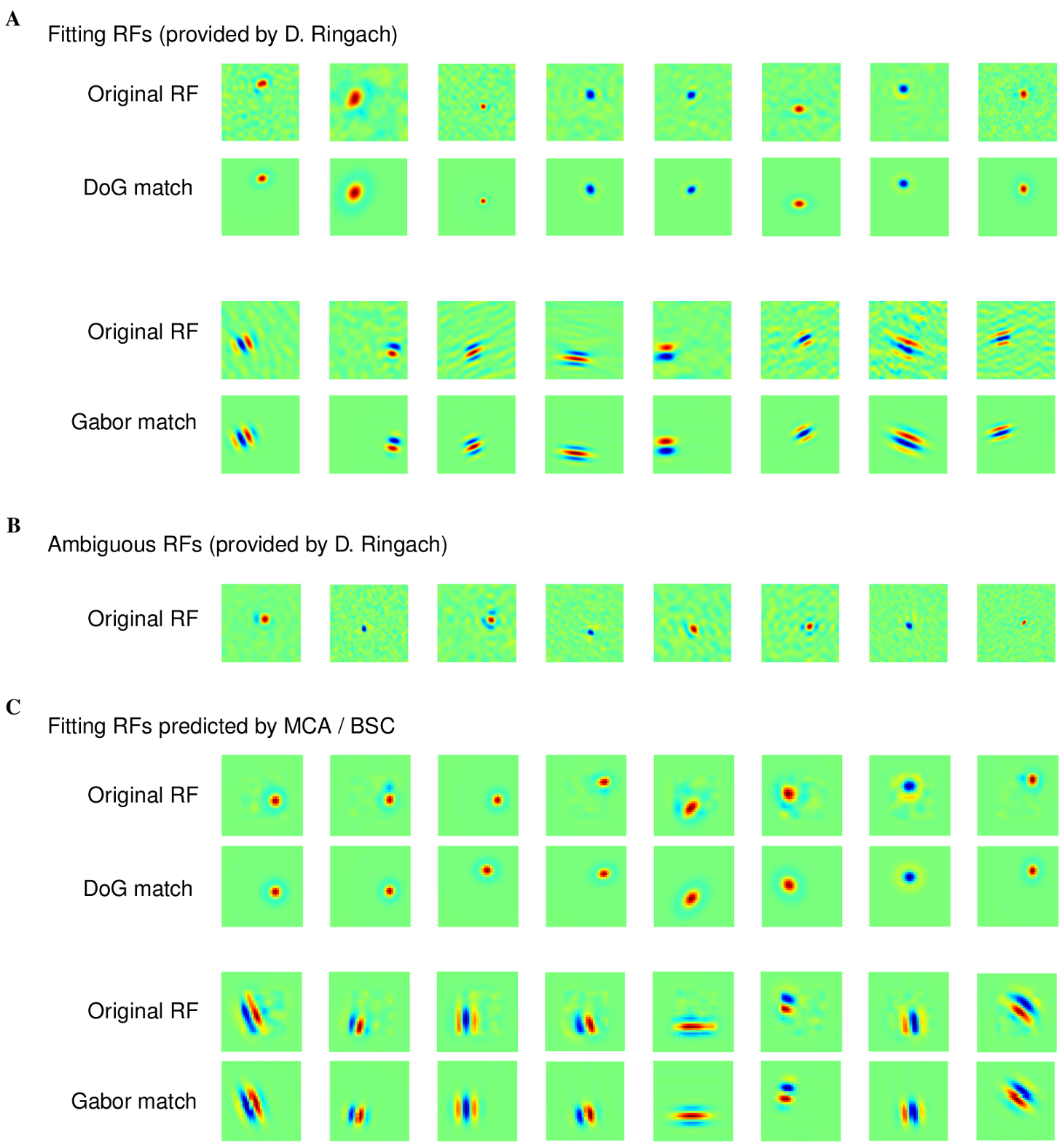

Supplement: Figure S7 — Fitting of learned and in vivo receptive fields with Gabor functions and DoGs. A Selection of 16 of the 250 receptive fields measured in macaque monkeys [15] using reverse correlation together with their resulting matches. A The upper row shows original recordings that were classified as globular, and the second row shows the corresponding DoG matches. The third row shows original recordings that were classified as Gabor-like, and the forth row shows their corresponding matches. B Examples of original receptive fields that were ambiguous, i.e., neither clearly difference-of-Gaussian nor Gabor-like. Note the Gaussian fields can be well matched by DoG and Gabor functions and are therefore inherently ambiguous. C A selection of 16 receptive field estimates resulting from numerical experiments. The fields and their matches are shown as in A. (TIFF) [file pcbi.1003062.s007.tiff]
